# A MUB E2 structure reveals E1 selectivity between cognate ubiquitin E2s in eukaryotes

**DOI:** 10.1038/ncomms12580

**Published:** 2016-08-23

**Authors:** Xiaolong Lu, Konstantin R. Malley, Caitlin C. Brenner, Olga Koroleva, Sergey Korolev, Brian P. Downes

**Affiliations:** 1 Department of Biology, Saint Louis University, 3507 Laclede Avenue, St Louis, MO 63103, USA; 2 Department of Biochemistry and Molecular Biology, Saint Louis University School of Medicine, 1100 S, Grand Avenue, St Louis, MO 63104, USA

## Abstract

Ubiquitin (Ub) is a protein modifier that controls processes ranging from protein degradation to endocytosis, but early-acting regulators of the three-enzyme ubiquitylation cascade are unknown. Here we report that the prenylated membrane-anchored ubiquitin-fold protein (MUB) is an early-acting regulator of subfamily-specific E2 activation. An AtMUB3:AtUBC8 co-crystal structure defines how MUBs inhibit E2∼Ub formation using a combination of E2 backside binding and a MUB-unique lap-bar loop to block E1 access. Since MUBs tether *Arabidopsis* group VI E2 enzymes (related to HsUbe2D and ScUbc4/5) to the plasma membrane, and inhibit E2 activation at physiological concentrations, they should function as potent plasma membrane localized regulators of Ub chain synthesis in eukaryotes. Our findings define a biochemical function for MUB, a family of highly conserved Ub-fold proteins, and provide an example of selective activation between cognate Ub E2s, previously thought to be constitutively activated by E1s.

## Abstract (web summary)

Regulators of the important ubiquitylation cascade are not well studied. Here, the authors report the crystal structure of a prenylated membrane-anchored ubiquitin-fold protein from *Arabidopsis* bound to an E2 protein and conclude that it is an example of selective activation between E2 enzymes.

## Graphical abstract

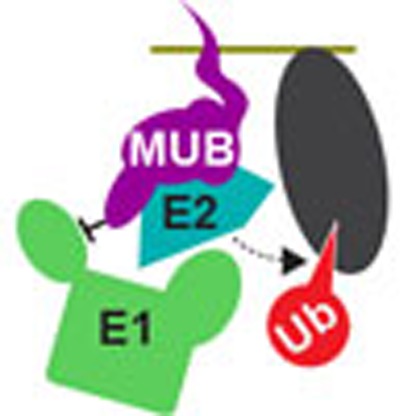

The covalent attachment of ubiquitin (Ub) to a protein substrate occurs through the sequential activity of Ub E1-activating, E2-conjugating and E3-ligating enzymes^1^,^2^, and has diverse regulatory functions in protein degradation, DNA repair, endocytosis and many other aspects of cell biology^3^,^4^. Each Ub must be activated by an E1, an 80–100 kDa protein composed of two E2 coordinating arms atop an activation body. The arms consist of the ubiquitin-fold domain (UFD) on one side, and the Cys domain, plus, first catalytic cysteine half domain on the other, bridged by the active adenylation domain (AAD) and inactive adenylation domain^5^. The AAD forms Ub-AMP using Mg^2+^-ATP and ubiquitin, which the Cys domain then acquires via a thioester linkage before rotating away to allow a second activation in the vacated AAD, resulting in E1(Ub)_2_ (ref. 6).

E1(Ub)_2_ is thought to recruit an E2 (refs 7, 8) and catalyse an E2 thioester linkage to Ub (E2∼Ub). Current models suggest the E1 UFD first interacts with the E2 in a distal conformation before rotating towards the opposed Cys domain to form a proximal configuration, placing the E1 and E2 active sites in range for Ub transfer^9^ (Fig. 1). This elegant mechanism affords regulation potential, which is indeed used between Ub-like protein tags (Ubls), but not traditionally within a Ubl family. For instance, Ubl E1-like enzymes for SUMO, Nedd8, and other Ubls do not activate Ub E2s, or vice versa^10^. In contrast, current models hold that the 37 diverse E2 enzymes of *Arabidopsis* are constitutively activated by either of two closely related E1s, UBA1 or UBA2 (refs 11, 12). The extent to which Ub E1s select Ub E2s is largely unexplored; although it would impact basal choices in the assembly of highly combinatorial ubiquitylation complexes.

In large part, Ub signalling outcomes are determined by the Ub-topology assembled on a given substrate^13^; for example, the addition of one Ub, or a Ub chain shaped by the use of seven internal Ub lysines^13^,^14^. Functionally, mono-ubiquitylation is associated with the endocytosis apparatus and DNA maintenance^15^,^16^, Lys48-linked Ub chains drive substrate degradation by the 26S proteasome^17^, and Lys63-linked Ub chains scaffold non-degradative protein complexes^18^. *In vitro*, E2s exhibit characteristic chain-building preferences that are strong for some E2 subfamilies such as Ube2S for Lys11-linked chains, Ube2K for Lys48-linked chains, Ube2N-Ube2V1 for Lys63 chains^19^, while the Ube2D subfamily requires additional guidance from an E3 (refs 20, 21).

In emerging studies, some E2s appear to control chain-building by scaffolding higher-order complexes through a non-covalent backside binding site (BBS) distant from the active-site Cys. For example, the BBS of Ubc13 positions the UEV (ubiquitin E2 variant) Mms2 to orchestrate K63 chain assembly^22^. Ube2D3 and the SUMO E2 Ubc9 use the BBS to interact with Ub or SUMO, to enhance chain elongation^23^,^24^, the BBS of Rad6 promotes Ub chain assembly unless blocked by Rad18 (ref. 25) and Ube2E3, which requires an intact BBS to add monoUbs^26^. Recent advances in understanding the HECT, RBR and APC E3s continue to reveal new E2 activities during the Ub-ligation cycle including unexplored avenues for Ub delivery into E3/substrate reactions and subsequent Ub chain-assembly processes^1^,^27^. Since E2s are dynamic members of the substrate modification complex, the nature of chain formation remains elusive^28^. How Ub chain topologies arise mechanistically, and whether the timing and positioning of E2 activation plays a role are key outstanding questions.

An interest in early regulation of the ubiquitylation axis drives our characterization of one representative family called membrane-anchored ubiquitin-fold (MUB) proteins. Although small at ∼120 amino acids, MUBs are unlike other ubiquitin-fold proteins including SUMO and NEDD8, because they do not have a C-terminal GlyGly motif for substrate attachment, and are probably not ubiquitin-like substrate modifiers. Instead, the MUB C-terminal CaaX box motif is prenylated and drives plasma membrane (PM) association in plants^29^. *Arabidopsis* MUBs1–6 interact specifically with the *Arabidopsis* group VI E2 family, and the single *Homo sapiens* HsMUB interacts only with the homologous Ube2D subfamily^30^. This E2 subfamily is a significant source of conjugating activity containing ∼20% of *Arabidopsis* E2 genes (7/37) and ∼10% of human E2 genes (3/24) and includes many examples with abundant expression *in vivo*^19^,^31^. The E2 subfamily is versatile, lacking Lys preference for chain-building, lacking N- or C-terminal extensions to the E2 core^21^ and promiscuous; functioning with all E3 families tested *in vitro* including HECT^32^, RING^24^,^33^, RBR^34^, SCF^35^ and APC^36^.

It was previously reported that MUBs function as PM adaptors for an E2 subfamily, but here, the analysis reveals that MUBs specifically inhibit activation of these critical Ub E2s. This unique finding led us to obtain a 2.8-Å co-crystal structure of AtMUB3 bound to AtUBC8. The structure reveals a mechanism for inhibition of E2∼Ub formation that discriminates between cognate Ub E2s. This establishes a previously unappreciated point of regulation in the combinatorial complexity of ubiquitylation. The E2 active site remains accessible in the AtMUB3:AtUBC8 structure and should play an integral role in ubiquitylation events near the PM.

## Results

### MUBs inhibit *Arabidopsis* Group VI E2 activation

**Figure 2:**
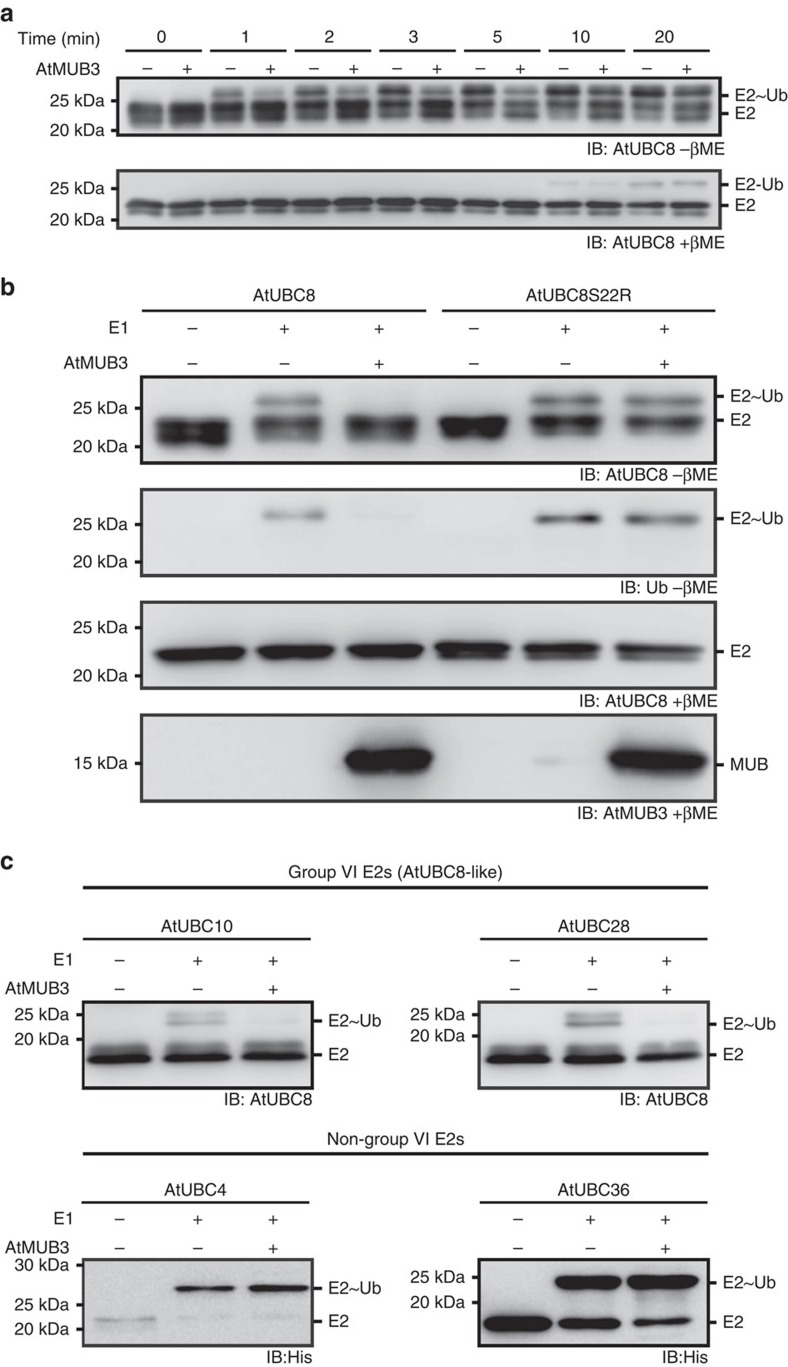
AtMUB3 specifically inhibits activation of AtUBC8-like E2s. (**a**) Thioester formation time course demonstrates the delayed charging of AtUBC8 in the presence of AtMUB3. Reactions were started with Mg-ATP addition, and stopped with Laemmli sample buffer at indicated time points and analysed by non-reducing (-βme, β-mercaptoethanol) and reducing (+βme) immunoblot (IB) with the indicated antibodies. (**b**) AtMUB3 inhibition of thioester formation was examined for AtUBC8 and AtUBC8^Ser22Arg^ using immunoblot (IB) analysis with AtUBC8, AtMUB3 or Ub antibodies. All samples contained Mg-ATP, Ub, reaction buffer and other reaction components as indicated. (**c**) Thioester-formation assays of representative Group VI E2s (top panel IB), and non-Group VI E2s (bottom panel IB). See also Supplementary Figs 9, 10 and 11).

To assess whether MUB binding regulates E2 function, we performed E2∼Ub thioester-formation assays on AtUBC8. For all time points tested, E2∼Ub formation was strongly reduced by AtMUB3 (Fig. 2a, top panel, −βme). AtUBC8-Ub peptide linkages, which could complicate the analysis, were observed after 10 min (Fig. 2a, bottom panel, +βme), so three minute reactions were used in subsequent assays. Ser22 on the BBS of AtUBC8 is distant from the active-site Cys85, and was previously determined to interfere with MUB binding^30^. E2∼Ub formation on AtUBC8^Ser22Arg^ was unaffected by AtMUB3, supporting the requirement of a direct interaction for inhibition (Fig. 2b). Since MUBs interact specifically with *Arabidopsis* group VI E2s, two additional subfamily members, AtUBC10 and AtUBC28 were tested. Significantly, AtMUB3 strongly inhibits their activation, however, AtUBC4 from group IV and AtUBC36 from group XV are unaffected (Fig. 2c). Collectively, MUB discriminates between cognate Ub E2s to inhibit the activation of a specific subfamily.

### Structure of the AtMUB3:AtUBC8 complex

**Figure 3:**
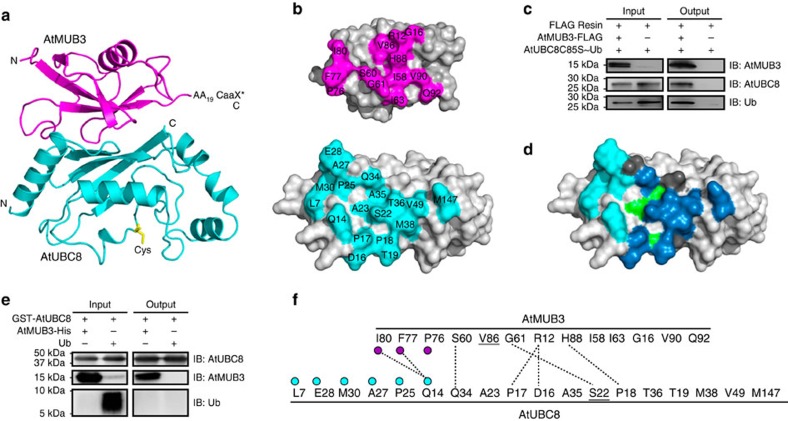
An AtMUB3:AtUBC8 co-crystal structure reveals extensive backside interaction that overlaps with non-covalent Ub binding. (**a**) Cartoon structure of the AtMUB3:AtUBC8 complex is coloured and labelled. AtMUB3 is coloured in magenta and AtUBC8 is coloured in cyan with its active-site Cys shown in yellow. The generalized position of the unstructured AtMUB3 carboxyl-terminus containing an additional 19 amino acids (AA_19_) and site used for protein prenylation (CaaX*) are indicated. (**b**) Interacting surface of AtMUB3:AtUBC8 complex is presented in open-book configuration. The binding residues are coloured magenta in AtMUB3 (top panel), cyan in AtUBC8 (bottom panel). (**c**) A FLAG pull-down assay between FLAG-AtMUB3 and AtUBC8^Cys85Ser^∼O∼Ub. The input and output proteins were visualized by immunoblotting (IB). (**d**) The AtMUB3 and Ub BBS interactions are mapped on the AtUBC8 surface. Unique binding for AtMUB3 and Ub are coloured in green and dark grey, respectively, while the overlap binding surface is coloured in dark blue. An additional AtMUB3-binding surface outside the canonical BBS is coloured in cyan. (**e**) A GST pull-down assay between GST-AtUBC8 and AtMUB3-His or Ub. (**f**) A diagram of AtMUB3:AtUBC8 interactions with contact residues listed for each molecule and dashed lines indicating salt bridges. Interacting residues outside of the BBS are indicated by magenta and cyan circles. Residues used to disrupt binding in previous studies are underlined. See also Supplementary Figs 2, 3, 4, 5, 12 and 13).

We framed two models for MUB competition against E1 with different downstream implications. First, that MUBs block E1 access to the E2 active site, which would imply that a MUB-bound E2 would neither be activated nor participate in downstream substrate ubiquitylation; and second, that MUBs block E1 association using a surface other than the E2 active site, supporting the possibility that MUB-bound E2s activated before joining the complex could participate in substrate ubiquitylation. To distinguish between these two models, we crystallized the AtMUB:AtUBC8 complex and obtained a 2.8-Å co-crystal structure (Table 1 and Fig. 3a). AtMUB3 occupies ∼900 Å^2^ of the AtUBC8 surface including the BBS and adjoining N-terminal residues, but does not encroach on the active-site Cys85 (Fig. 3b). Indeed, AtMUB3 pulls-down AtUBC8^Cys85Ser^ with Ub linked to the active site through an oxyester bond (Fig. 3c). Importantly, AtUBC8 Ser22 forms a salt bridge with AtMUB3 Gly61, central to additional salt bridges and extensive hydrophobic interactions (Fig. 3b,f) consistent with the submicromolar dissociation constant K_d_ as measured by isothermal titration calorimetry (ITC, Supplementary Fig. 1) and enzymatic assays^30^ (Fig. 2b). Comparing AtUBC8 with Human Ube2D3 (3UGB) 37, which is 80% identical and 89% similar at the amino acid sequence level, reveals an r.m.s.d. of 0.7 Å for 146 Cα atoms suggesting that AtUBC8 does not undergo a large MUB-induced conformational change (Supplementary Fig. 2).

In the complex, AtMUB3 bears strong resemblance to known Ub-fold structures, featuring a central β-sheet cradling a major α-helix, and a second minor α-helix residing near the N-terminus. Excluding the connecting loops and the unstructured C-terminus, AtMUB3 aligns closely with Ub, r.m.s.d. 1.8 Å for 71 Cα atoms (2FUH)^24^, and SUMO, r.m.s.d. 1.6 Å for 74 Cα atoms (2UYZ)^38^. There are no MUB crystal structures for comparison, but the Ub-fold core of AtMUB3 aligns well with an NMR structure of AtMUB1 in solution, r.m.s.d. equivalent 0.8 Å (1SE9) for 66 Cα atoms^39^. Yet there are significant differences in loop positions and flexibility (see detailed description below) between the AtMUB1 and AtMUB3 structures despite strong sequence conservation of 46% identity and 68% similarity (Supplementary Fig. 2). Consistent with efficient MUB prenylation^29^, the C-terminal nineteen residues of AtMUB3 are unstructured, suggesting a flexible tether to the C-terminal Cys115, which is converted to a C-terminal geranylgeranyl carboxymethyl Cys *in planta* and used for PM-anchoring. The model that emerges is a MUB-anchored E2 with its active site exposed to cytosolic PM proximal proteins, yet with E2∼Ub turnover biochemically suppressed possibly to guide E2 chain-building preferences of the E2 subfamily.

### MUB uses the Ub E2 BBS for interaction

To define a mechanism for AtMUB3 inhibition of E2∼Ub formation, we examined known functional E2 surfaces, most obviously, the non-covalent BBS. In detail, the MUB BBS interaction uses the AtUBC8 α1β1-loop (Asp16, Pro17, Pro18, Thr19), β1-strand (Ser22, Ala23), β2-strand (Gln34, Ala35, Thr36 and Met38), β3-strand (Val49) and the C-terminal residue Met147. Meanwhile, AtUBC8 interacts with AtMUB3 β1-, β2-, β4-, β5-strands (Arg12, Gly16, Ile58, Val86, His88 and Val90), β4α2-loop (Ser60, Gly61 and Ile63) and one residue on the C-terminal tail (Gln92; Fig. 3b,f and Supplementary Fig. 3). The surface-forming residues are highly conserved in this family of E2s from plants, fungi and humans. These residues are not conserved in other major *Arabidopsis* E2 groups, revealing why MUBs are specific to the Group VI subfamily (Supplementary Figs 4 and 5).

Comparing AtMUB3:AtUBC8 with the most related structure, Ub:Ube2D3 (2FUH)^24^, reveals that MUB interacts with E2s using a similar configuration (Supplementary Fig. 3), albeit with several critical differences. MUB and Ub interact with the BBS using nine analogous residues. Each protein also has three unique interaction residues. For Ub, AtUBC8-analogous residues Ser20, Val26 and Leu51 (Fig. 3d, dark grey) would pull Ub towards the E2 C-terminus, whereas AtMUB3 unique interactions using Pro18, Ala23 and Ala35 tip AtMUB3 towards the AtUBC8 N-terminus (Fig. 3d, green). Collectively, the MUB to E2–BBS surface specifies and anchors the interaction, but has no clear role in inhibition of E2∼Ub formation, and so we turned our attention to the adjacent interaction surface.

### A MUB lap-bar loop extends beyond the E2–BBS

Detailed examination of MUB binding outside of the Ub-analogous BBS reveals a predominant role for the AtMUB3 α2β5-loop, referred to, from here on, as the lap-bar loop (LBL). The LBL reaches from the BBS towards the E2 N-terminus to occupy an additional ∼290 Å^2^ surface, highlighted in cyan in Fig. 3d,f. It is likely that the increased area accounts for the significantly stronger interaction that we detect experimentally, relative to E2 and Ub. For instance, the AtUBC8 BBS is an efficient bait for MUB pull-down, but not for Ub pull-down (Fig. 3e). Similarly, we have routinely detected the interaction of MUB and E2 by yeast two-hybrid, but have never detected a Ub E2 interaction^30^. In addition, previous reports of both Ub and SUMO BBS interactions have used elegant structural methodologies, but to our knowledge have not successfully used pull-downs or yeast two-hybrid techniques to demonstrate interaction^21^,^23^,^40^,^41^,^42^. Further analysis revealed a more negative ΔG for MUB:E2 compared with other known BBS interactions (Supplementary Table 1). These results suggest that the LBL enhances the stability of the MUB:E2 interaction.

**Figure 4:**
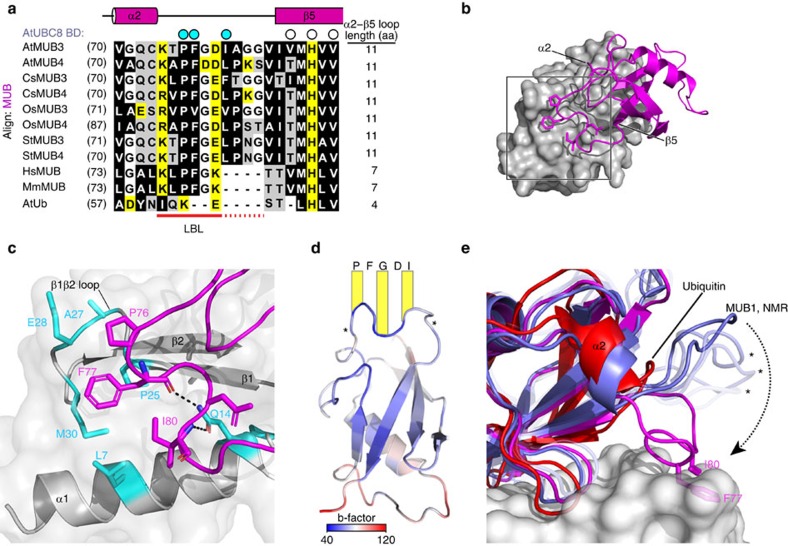
The MUB LBL is stabilized by E2 surface interaction. (**a**) Structure-based sequence alignment for the LBL region of the AtMUB3/4 protein family from *Arabidopsis*, *Arabidopsis thaliana* (At); cucumber, *Cucumis sativus*; rice, *Oryza Sativa* (Os); potato, *Solanum tuberosum* (St) and the single MUB proteins from human, *Homo sapiens* (Hs) and mouse, *Mus musculus* (Mm). Ubiquitin is included for comparison. Residues in direct contact with E2 (AtUBC8 BD) are indicated by circles above the alignment, among which, cyan circles indicate LBL residues and open circles indicate BBS-binding residues. The most universally conserved part of the LBL is underlined in red, while plant-specific conservation is underlined with red dots. The aligned amino acids are coloured black (hydrophobic), yellow (charged) and white (polar) and the length of the α2β5-loop is indicated to the right. (**b**) Structure of the AtMUB3 LBL is highlighted by a square, with AtUBC8 in surface structure (grey) and AtMUB3 in cartoon structure (magenta). Secondary structures flanking the LBL in AtMUB3 are indicated. (**c**) Detailed view of the AtMUB3 LBL and AtUBC8 with interacting residues rendered with sticks. LBL (magenta) and AtUBC8 (grey) with residues coloured in cyan. Salt bridges are shown as dashed lines. (**d**) b-factors are plotted on AtMUB3 (cartoon) viewed from the E2 perspective. Core residues of the bar are listed and flexible side arms are indicated with asterisks. (**e**) Superposition of Ub (red) modelled from a backside Ube2D3-binding structure, AtMUB1 alone in solution (purple blue) and the AtMUB3 (magenta):AtUBC8 (grey, surface) complex. LBLs in different states of the AtMUB1 NMR structure (asterisks) exhibit flexibility and various ‘open' conformations, while LBL residues in the AtMUB3 structure (sticks) are coordinated by the E2 surface demonstrating a ‘closed' conformation.

Compared with Ub, the MUB-unique LBL has expanded by 3 and 7 residues in animals and plants, respectively (Fig. 4a). In cases examined, the LBL contains the consensus (Lys/Arg)xProPheGly((+)/(−)) motif. The larger plant LBL follows with (Ile/Leu/Phe/Val) that contributes hydrophobic contacts and a main chain salt bridge to AtUBC8 (Figs 3f and 4a). In all cases, the invariant Pro and highly conserved Phe are the core of the LBL:E2 interaction. Adjacent to the MUB Ub-fold BBS interaction, the LBL loop flattens and conforms to the AtUBC8 surface (Fig. 4b). A detailed view of the interaction reveals how the LBL nestles into a hydrophobic AtUBC8 pocket contacting the β1β2-loop residues Pro25, Ala27, Asp28, Met30 on one side and the N-terminal α-helix residue Leu7 on the other (Fig. 4c). Gln14, also on α1, stabilizes the interaction forming salt bridges to the Phe77 and Ile80 main chain positions, possibly contributing to the distinctive sigmoidal shape of the distal ‘bar' of the LBL. When bound to AtUBC8, the bar is rigid, but flanked by flexible segments indicated by the b-factor values of the structure (Fig. 4d). Superposition with Ub reveals how extension of the MUB LBL contacts the hydrophobic E2 patch (Fig. 4e).

Comparison of free AtMUB1 and the AtMUB3:AtUBC8 complex revealed significant movement of the LBL on complex formation. In the AtMUB1 NMR structure, the LBL adopts flexible conformations in close proximity to the core Ub-fold. In complex, however, the LBL moves away from the core to interact with the AtUBC8 α1-helix and the β1β2-loop. This conformational change is significantly larger in amplitude than the movement of this loop in the NMR structure. Specifically, the AtMUB3 Phe77 sits in the E2 pocket, however, the corresponding AtMUB1 residue is highly mobile moving over an 8.5-Å span, but never coming within 5.4 Å of the AtMUB3 Phe77 position (Fig. 4e). Small differences occur in the conformation of the short α2 helix that leads into the LBL and the conformation of β2-strand at the other end of the LBL (Supplementary Fig. 2). Thus, MUB undergoes a conformational change to snap the LBL onto the E2 surface.

### MUB inhibition is conserved between plants and humans

**Figure 5:**
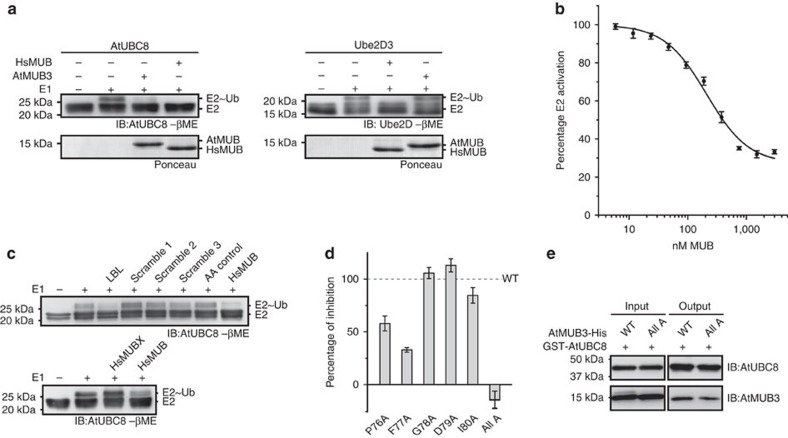
Functionally conserved MUB LBL is sufficient for inhibition of E2∼Ub formation. (**a**) Thioester-formation assays, as described in Fig. 2, of AtUBC8 (left) and Ube2D3 (right), each exposed to both *Arabidopsis* and human MUBs. (**b**) Using HTRF–FRET, Ube2D3 activation was monitored with indicated concentrations of HsMUB to establish an inhibition curve. The initial velocity was linear over the first 10 min and is represented as percent E2 activated, with 100% velocity taken in the absence of MUB and 0% in the absence of ATP. Plot represents three independent assays±s.e.m., fit to the one-site binding equation. An additional three assays fit to the Hill equation with coefficient *n* produced nearly identical results with *n*∼1. See also Supplementary Fig. 1. (**c**) Top panel, thioester-formation assays of AtUBC8 exposed to N-LKLPFGKT-C (HsMUB LBL), three scrambled peptides or an amino acid mixture equimolar to the human LBL (AA control). Bottom panel, thioester-formation assays of UBC8 exposed to HsMUB chimeric for the Ub α2β5-loop (HsMUBX). (**d**) Quantification of AtMUB3 LBL mutant protein inhibition was performed by normalization to WT as 100% (dashed line),±s.e.m. and *n=3* for all data points (Supplementary Figs 6 and 7). AtMUB3 with the LBL core converted to alanines is P76A F77A G78A D79A I80A (All A). (**e**) An interaction study between AtUBC8 and AtMUB3 All A LBL mutant proteins was carried out using GST pull-down assays. The input and output proteins were visualized by immunoblotting (IB) as indicated. See also Supplementary Figs 14, 15 and 16).

To test whether the differences between plant and animal LBLs influence their activity, we compared them in reciprocal inhibition experiments. The human homologue of MUB, HsMUB, reveals potent inhibition of not just Ube2D3∼Ub formation but also AtUBC8∼Ub formation, suggesting that the smaller HsMUB LBL is sufficient. AtMUB3 does not inhibit Ube2D3∼Ub, suggesting that the four residue expansion of plant-specific LBLs prohibits access to the Ube2D3 surface (Fig. 5a).

### MUB inhibition occurs at physiological concentrations

To determine the magnitude of MUB inhibition, we investigated HsMUB inhibition of Ube2D3 using an independent method based on Homogeneous Time Resolved Fluorescence–Förster Resonance Energy Transfer (HTRF–FRET). In this assay, fluorescein-labelled Ub behaves as a FRET acceptor once the thioester is formed with biotinylated E2 labelled with a streptavidin-terbium FRET donor. This assay establishes a K_i_ of 220±16 nM, and demonstrates that up to 70 per cent of Ube2D3 activation can be inhibited (Fig. 5b). These results are highly consistent with the PAGE-based assays where we typically see low levels of E2 activation even in the presence of saturating MUB. Taken together, they indicate MUB inhibition of E2∼Ub formation is conserved and broadly relevant at physiological MUB concentrations in organisms that use the ubiquitylation system.

### The LBL interferes with E2 charging

To further test the prediction that LBL is sufficient for inhibition, we applied a minimal human LBL (LeuLysLeuProPheGlyLysThr) peptide to E2∼Ub-formation assays. This peptide has potent inhibitory activity when added to assays at 5 mM, similar to 30 μM complete HsMUB. Three scrambled peptides (5 mM, each), or eqi-molar free amino acids have no effect. In a complementary experiment, HsMUB with the LBL replaced with the Ub α2β5-loop, named HsMUBX, completely loses the ability to inhibit E2∼Ub formation (Fig. 5c). Further, AtMUB3 with the core LBL residues ProPheGlyAspIle all converted to alanines fails to inhibit, while retaining binding in pull-down assays. Individual mutations of this sequence reveal that the highly conserved ProPhe motif is most responsible for inhibition (Fig. 5d,e and Supplementary Figs 6 and 7).

### E1 and MUB require access to the same E2 residues

**Figure 6:**
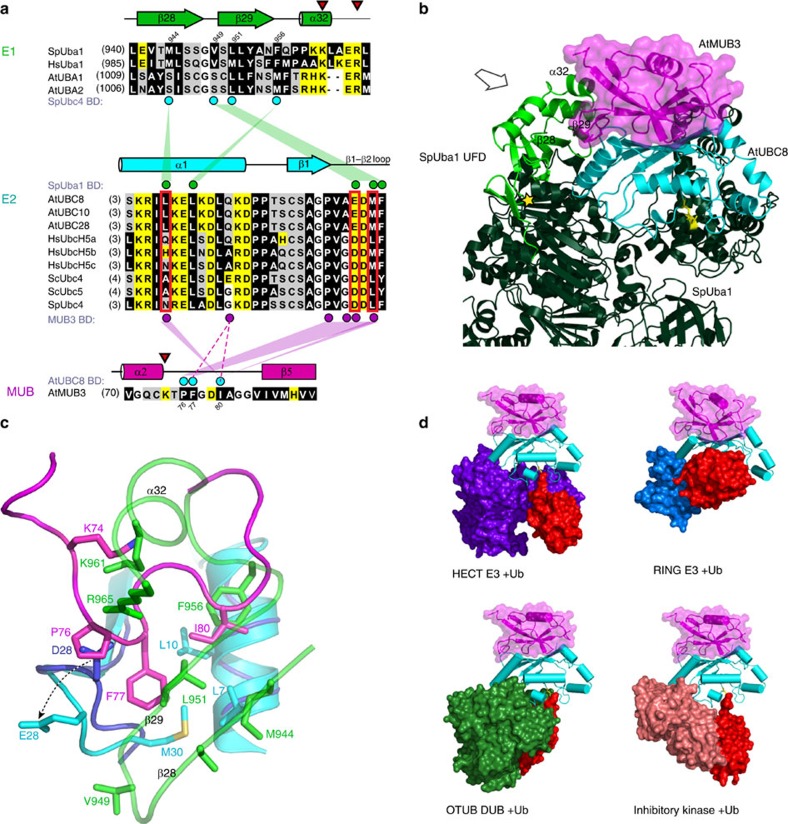
The MUB LBL conflicts with E1 E2 association. (**a**) Structure-based sequence alignment for E1s (ref. 58) in *Arabidopsis thaliana* (At), *Homo sapiens* (Hs) and *Schizosaccharomyces pombe* (Sp) focused on the UFD E2 interacting region. Cyan circles indicate residues that are most critical for E1 UFD:E2 docking and E2∼Ub formation^9^. A structure-based sequence alignment for E2s (UBCs) in At, Hs, Sp and *Saccharomyces cerevisiae* (Sc) focused on the region that interfaces with the MUB LBL. Residues in contact with E1 UFD and MUB are indicated by circles above and below the alignment, respectively. Magenta circles indicate residues that interact with MUB LBL. Green circles indicate residues that are most critical for E1 UFD docking^9^. Residues that contact both MUB and E1 are indicated with red. The alignments are coloured as described in Fig. 4. Lines between E1, E2 and MUB indicate structurally determined interactions. Red triangles indicate spatially conflicting residues. (**b**) Structural superposition model of E1, E2 and MUB. AtMUB3:AtUBC8 complex (in magenta and cyan) was superimposed to the SpUba1:SpUbc4:Ub:Mg-ATP complex using E2s for alignment. SpUba1 is featured in dark green with UFD highlighted in light green, while the Ub, SpUbc4 and Mg-ATP are not shown (PDB code 4II2). Secondary structures for Uba1 are labelled. Arrow indicates perspective for **c** detail. Yellow star indicates the UFD hinge. (**c**) Detailed cartoon structure of MUB LBL region (magenta) and E1 UFD (green) conflict region near the E2 N-terminal α-helix (AtUBC8 in cyan, SpUbc4 in dark blue, arrow indicates loop displacement in the current structure). The critical residues involved in contact and conflict are labelled and shown as stick representations. (**d**) No additional conflicts were obvious in structure models of the AtMUB3:AtUBC8 complex (magenta, cyan, respectively) docked to various Ub system Ube2D3 containing complexes constructed via E2 structure alignments. Structure models include portions of a HECT E3, NEDD4L HECT Domain (3JVZ)^32^; a RING E3, BRIC7 RING domain (4AUQ); a deubiquitinating enzyme, OTUB1 (4LDT)^59^; and a prokaryotic inhibitory kinase, OSPG (4BVU)^60^, which are coloured purple blue, marine, forest green and deepsalmon, respectively. Ub is coloured in red.

To better define the mechanism used by the LBL to inhibit E2∼Ub thioester formation, we compared the LBL:E2 surface and a previously characterized E1:E2 surface (PDB code 4II2), which reveals that LBL interferes with E1:E2 interaction. The *S. pombe* E1 (SpUba1) uses the conserved UFD domain to coordinate E2(SpUbc4)∼Ub formation. The interface is formed by SpUba1 β28, β29 and α32, and includes two key E2-binding residues, Leu951 and Phe956 in *S. pombe*, which are conserved in *Arabidopsis* UBA1/2 as Leu1020 and Met1025 (Fig. 6a, top). Mutation of these SpUba1 residues decrease E2∼Ub formation by at least 70 percent^9^. This is strikingly similar to the decrease observed with saturating MUB (Fig. 5b).

Notably, plotted in Fig. 6a, middle, the N-terminal twenty-nine E2 residues of the α1 helix through the β1β2-loop contain residues for both (i) E2∼Ub formation^9^ and (ii) LBL binding (Fig. 4c). Positions Leu7, Glu28 and Met30 are key contacts for Phe77 and Ile80 of AtMUB3 LBL, and Leu951 and Phe956 of the SpUba1 UFD, respectively (magenta versus green circles, Fig. 6a). Most of the conflict occurs in two focal regions, the C-terminal half of the α1-helix and the β1β2-loop, which form a conserved contiguous surface in both AtUBC8 and SpUbc4 structures.

To examine the conflict-implicated residues in three dimensions, a model was generated superimposing the AtMUB3:AtUBC8 complex onto the SpUba1:SpUbc4 structure (Fig. 6b), which reveals that the conflict is exclusive to the UFD of the E1. Here, the Phe77 and Ile80 of the LBL are imposed directly into the hydrophobic groove used by SpUba1 residues Leu951 and Phe956 (Fig. 6c). The central position of Phe77 in the conflict explains the strong loss of inhibition seen with Phe77Ala (Fig. 5d). Aside from Phe77, Pro76 appears to play a key role positioning these residues in the conflict zone. Taken together, the E1:MUB:E2 model reveals direct conflicts between the E1 residues required for efficient E2∼Ub formation and the MUB LBL residues found to inhibit E2∼Ub formation, and in conjunction with the BBS interaction explains why MUBs can specifically inhibit the E2 subfamily.

### MUB does not sterically interfere with downstream enzymes

**Figure 7:**
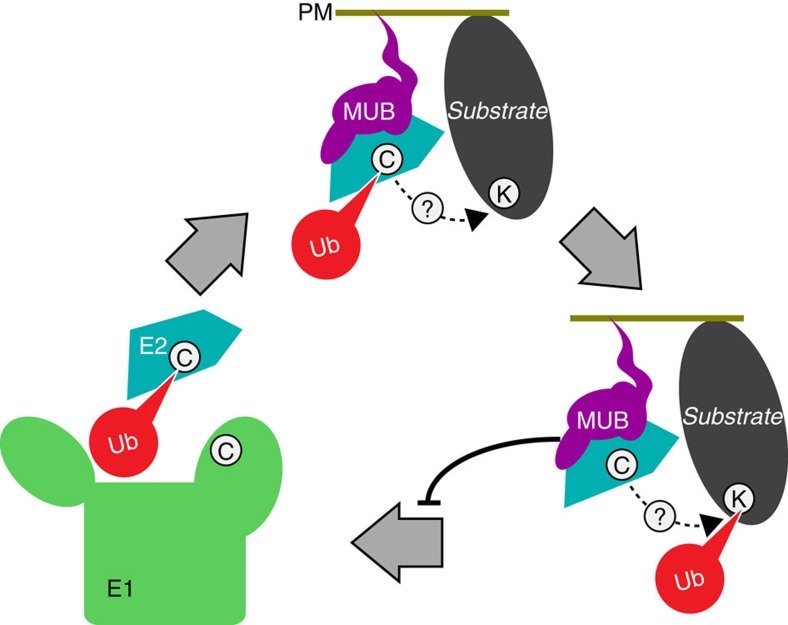
A model: MUB inhibition of E2∼Ub formation. MUB-regulated E1 charging of E2 near the PM.

In contrast to the E1 conflict, we were unable to model any obvious MUB challenge to other classes of Ub family Ube2D-interacting proteins including HECT E3s, RING E3s, OTUB DUBs or even pathogen-derived inhibitory kinases, suggesting that MUB-bound E2∼Ub should participate normally in the conjugation pathway until requiring reactivation (Figs 6d and 7). Nonetheless, these models could benefit from direct biochemical investigation or high-resolution structural analysis. At this time there are many possible allosteric effects on the E2 that we cannot rule out, which merit further study.

## Discussion

The main finding of this work is that MUB exerts high-level regulation on the ubiquitin system in plants and mammals by inhibiting E1 activation of an important E2 subfamily (Fig. 2). E2 activation is required for all subsequent ubiquitylation events^1^; however, it has not been previously documented as a point of regulation. These studies provide a functionally distinguishing characteristic for the E2 family as a whole; whether a subfamily is susceptible to MUB inhibition when near the PM or not. Moreover, the identification of this regulatory mechanism has broad implications for the ubiquitylation system because of the critical E2 clade affected. These E2s include *Arabidopsis* Group VI and human Ube2D, which are abundant, highly conserved in eukaryotes and implicated in chain initiation^19^,^43^. Considering the E1 interaction, the inhibition caused by MUB gives new perspective on UFD surface requirements for E2 activation.

A mechanistic definition of the inhibition we have observed was made possible by a 2.8 Å structure of AtMUB3:AtUBC8, which revealed that MUB differentially recruits Ub E2s using the BBS (Fig. 3). The MUB E2–BBS interaction provides a fourth different Ub or Ubl BBS combination implicated in E2 regulation (Supplementary Fig. 3), which currently includes Ub, SUMO and Nedd8 (refs 23, 24, 40, 41, 44). However, unlike these proteins, MUB is not a protein modifier and thus its position on the BBS likely exerts native regulation of the Ub E2 by occluding Ub access, which would have ramifications for chain building and reaction dynamics. This notion is supported by Rad18, and Ube2D3^Ser22Arg^ suppression of polyubiquitylation^21^,^25^. However, studies of chain assembly are beyond the scope of this study, since it is critical to first understand inhibition of E2∼Ub formation by a MUB E2 complex.

Further structural analysis reveals that MUB suppresses E2 activation using the unique LBL (Figs 4 and 5). The LBL disrupts E1 UFD interaction with the E2, providing an explanation for the inhibition of E2∼Ub formation (Fig. 6). To our knowledge, the control of E1 access to a Ub E2 subfamily, reported here for MUB, is unique in the current literature.

It is worth noting that unlike plants^12^, vertebrates and sea urchins express a second evolutionarily divergent Ub E1 called Uba6 (ref. 45) that has inherent specificity for a Ub E2. While Uba1 and Uba6 both activate the Ube2D subfamily, only Uba6 activates the E2 Use1 (ref. 46). Ube1/Uba6 chimeric enzymes revealed that E2 preference resides in the UFD domain^45^. This is reminiscent of MUB manipulation of the UFD interaction surface to block E2 activation. Yet, Ube1 and Uba6 have hard-wired UFD E2 specificities, while MUBs conditionally obscure UFD access for E2. Specifically, MUB inhibition is dependent on subcellular localization and the ability to recognize the BBS. Another difference from Uba6 is that MUBs are more broadly distributed; as they are found in all eukaryotes except for a select few *Ascomycota* including brewer's yeast.

Ultimately, examination of downstream reactions will be required to establish how MUB affects the activated E2 population. In large part, the physiological acceptor for a MUB:E2 donated Ub will determine the contribution of Ub discharge rate to the equilibrium of charged Ub on MUB:E2. However, as opposed to the clear structural evidence for MUB impedance of E1 association with E2, we do not detect a conformational change in the E2 active site. Yet, this complex issue has precedents and clearly warrants additional investigation. For example, the G2BR domain of the RING E3 gp78 contacts the BBS of Ube2g2 to effect allosteric changes of the E2 active site^47^,^48^, as does interaction with Cue1p^49^. Furthermore, mutations of E2 UFD-interacting residues alter E2 conjugation activity even when activation occurs via E1 lacking its UFD domain^50^.

Structurally, it is clear that the prenylated C-terminus of MUB is not obstructed when a MUB is E2-bound, affirming how a MUB:E2 complex localizes to the PM. Furthermore, MUB does not obscure the E2 active-site Cys or downstream ubiquitylation factors (Fig. 6). Thus, MUB:E2∼Ub should be available for target protein modification.

Our current working model is that charged E2s bind to MUBs, but then, following Ub discharge, experience slow reactivation due to the competitive inhibition of E1 introduced by MUB:E2 binding. This model is based on the kinetic analysis demonstrating that even with saturating MUB, ∼30% E1 activity remains uninhibited (Fig. 5b). Thus, E1 can still activate MUB-bound E2, but at a much reduced rate (K_cat'_), compared with the native E1 activation rate (K_cat_) for free E2 (Supplementary Fig. 8). It is also possible that E2 needs to dissociate from MUB before reactivation. This raises the interesting question of how a predicted MUB:E2 target modification cycle would provide energy to eject a spent E2. In any case, it is currently clear that MUB would introduce a severe limit to Ube2D reactivation rate for reactions occurring near the plasma membrane.

MUB:E2 participation in a ubiquitylation reaction would add two unique properties: (i) the unavailability of the E2–BBS, and (ii) the obstruction of the E1-binding site, both forces that suppress chain formation, raising interesting possibilities for Ub chain formation near the cell surface. Here, the current gap in knowledge regarding processive Ub chain formation allows measured speculation^1^,^27^. One proposed framework for the chain-building process is that the difference between mono- and poly-ubiquitylation is largely determined by E2 turnover rate^28^ where slow turnover promotes mono-ubiquitylation. On the basis of our data we predict that MUBs will decrease the turnover rate of the E2s that they recruit to the membrane. Interestingly, in plants there have recently been a number of important monoubiquitylated PM proteins described, including BORON TRANSPORTER1 (ref. 51), IRON-REGULATED TRANSPORTER1 (ref. 52), PRR FLAGELLIN-SENSING2 (ref. 53), PIN-FORMED 2 (ref. 54) and PHOTOTROPIN1 (ref. 55). These and other proteins localize to the PM, where MUBs are found and mono-ubiquitylation is prevalent. The current findings suggest that E2 activation is regulated with subcellular precision, however, key future questions include what substrates are MUB complex-modified, and with what Ub-topology. These questions warrant continued study to better understand selective Ub E2 activation by E1s in eukaryotes.

## Methods

### Cloning and mutagenesis

The coding regions of AtMUB3 and HsMUB were PCR-cloned to remove stop codons, enable pET28b ligation and append C-terminal 6 × HIS tags. C-terminal 6 × His plus FLAG-tagged AtMUB3 were generated by DNA 2.0. Generation of N-terminal 6 × HIS AtMUB3, GST-AtUBC8 and AtUBC8^Ser22Arg^ was as described^30^. AtUBC8^Cys85Ser^ and all AtMUB3 mutants were generated by bridging-PCR and PCR-cloned into pET28b.

PCR-cloning of AtUBCs 4, 8, 10, 28 and 36 with intact stop codons, and AtUBC8, AtUBC8^Ser22Arg^ and Ube2D3 lacking stop codons were moved into pENTR-D-TOPO for subsequent LR cloning. AtUBC8, AtUBC8^Ser22Arg^ and Ube2D3 were transferred to p0GWA^56^ to generate C-terminal 6 × HIS E2 constructs, while AtUBCs 4, 8, 10, 28 and 36 were transferred to pHGWA^56^ to generate N-terminal 6 × HIS E2 constructs. All constructs were sequence verified and catalogued as glycerol stocks for distribution. Primers for construct assemblies are listed in Supplementary Table 2.

### Protein expression and purification

Throughout, protein concentrations refer to total protein. All proteins were expressed in Rosetta 2 (DE3) pLacI *E. coli* (Novagen). Cultures in LB medium were induced by addition of 0.2 mM isopropyl-β-D-1-thio-galactoside at 16 °C for 16 h with vigorous shaking. For activity assays, AtMUB3 and UBC preparation cell pellets were sonicated in 50 mM Tris-HCl, pH 7.5, 300 mM NaCl, 10% Glycerol and 10 mM Imidazole. The soluble lysate fraction was purified on TALON metal affinity resin (Clontech) washed sequentially in sonication buffer plus 20 mM then 40 mM Imidazole, and eluted using 200 mM Imidazole. For crystallization, AtMUB3 cell pellets were sonicated in 25 mM HEPES, pH 7.5, 500 mM NaCl, 10% glycerol, 1 mM TCEP, 0.4 mM CHAPS and 10 mM Imidazole, washed and eluted as above, and then applied to hydroxylapatite resin made in lab according to ref. 57. Hydroxylapatite flow-through was incubated at 70 °C for 10 min and centrifuged to remove heat-labile contaminants. For AtUBC8 crystallography, activity-grade elutions were diluted 10-fold and re-purified using metal affinity.

### *In vitro* interaction studies

GST-tagged AtUBC8 was induced as described above, and the soluble extract fraction was immobilized on GSH resin (Thermo Scientific) for 20 min, followed by three washes with sonication buffer. Equimolar His-tagged AtMUB3, AtMUB3 mutants and Ub were incubated separately with AtUBC8-loaded GSH resin for 15 min, washed three times using sonication buffer and boiled in Laemmli buffer. MUB recovery was determined by immumoblotting and chemiluminescent detection using Fujifilm LAS4000 imaging.

For FLAG pull-down assays, His-tagged AtUBC8^Cys85Ser^∼O∼Ub was generated^40^. Flag-tagged AtMUB3 was immobilized on FLAG resin (Thermo Scientific) for 20 min, followed by three washes with sonication buffer. AtUBC8^Cys85Ser^∼O∼Ub protein or Ub were incubated separately with AtMUB3-loaded FLAG resin for 15 min, washed three times using sonication buffer and boiled in Laemmli buffer. For immunoblotting, antibodies against Group VI E2s, MUBs and GST were custom made and used at 1:5,000, Anti-FLAG (Sigma) and anti-Ub (Boston Biochem) were used at 1:2,000). Immunoblot HRP luminescence was imaged using a FujiFilm LAS4000.

ITC was performed on 8 μM UBC8 with 110 μM MUB3 as the titrant using a MicroCal iTC200 (GE). Proteins were purified as described for crystallization and dialyzed extensively against a buffer containing 25 mM HEPES, pH 7.5, 200 mM NaCl, 1 mM TCEP. Data were processed with MicroCal Origin 7 software, and normalized against the dialysis buffer's heat of dilution.

### Crystallization

An equimolar mixture of AtMUB3 and AtUBC8 was gently mixed, concentrated to 12 mg ml^−1^, and exchanged to 25 mM HEPES, pH 7.5, 500 mM NaCl, 1 mM TCEP and 0.1 mM CHAPS using Amicon Ultra-15 centrifugal filter (Millipore). Crystals grew using hanging-drop vapour diffusion at 20 °C when mixing equal volumes of protein complex with a well solution of 2 M ammonium sulfate. Six-sided bi-pyramidal crystals with a tall and short apex appeared within 1 day and grew to full size within 4–7 days. Crystals belong to hexagonal space group P6_3_22 with unit cell parameters a=b=135.7 Å, c=202.1 Å. Final data set with resolution of 2.8 Å was collected on the GM/CA-CAT 23ID-D beam line at APS, ANL. ScUbc4 (1QCQ; PubMed: 8268156) and ubiquitin structure (3NOB/A; PubMed: 20655260) were used for molecular replacement using Phaser (PMID: 19461840). Model building and refinement were completed using the Refmac and Coot programs (PMID: 21460454; PMID: 15572765). Final model include AtUBC8 amino acids 1–147 in chain A and 1–148 in chain C, and AtMUB3 amino acids 2–94 in chain B and 3–94 in chain D with amino acids 2, 3, 43 of chain B and 3–5, 43, 53, 68 of chain D modelled as alanines due to poor density. Data collection and refinement statistics are shown in Table 1.

### E2 thioester-formation assays

E2∼Ub thioester-formation assays were performed at 25 °C using 0.25 μM E1, 3 μM E2, 12 μM Ub and 50 mM HEPES, pH 8.0, initiated with the addition of 1 mM Mg-ATP. When included, 30 μM AtMUB3 was pre-incubated for 15 min at 25 °C. Reactions were terminated by the addition of non-reducing or reducing Laemmli buffer. Protein samples were visualized and analysed as described above, and quantified using FujiFilm Multi Gauge software. Relative inhibition efficiency was determined by comparing the inhibition efficiency of AtMUB3 mutants with wild type as follows; relative inhibition efficiency=(1−(E2∼Ub with AtMUB3 mutant/E2))/(1−(E2∼Ub with WT AtMUB3/E2)).

HTRF–FRET E2-activation assays were performed using reagents adapted from the E1/E2 Lite kit (LifeSensors, Malvern, PA). Briefly, assays contained 1 nM E1 (HsUBE1), 10 μM Mg-ATP, 50 nM Ub-fluorescein, 0.9 nM Streptavidin-terbium and 5 nM biotinylated E2 (Ube2D3) in 50 mM Tris-HCl, pH 7.5, 5 mM MgCl_2_, 0.05% CHAPS reaction buffer. HsMUB was exchanged into reaction buffer, and combined with E2 at the indicated concentrations for a 15 min pre-incubation in a 30 μl volume. Addition of 15 μl E1 started the reactions, while controls lacking ATP established the baseline. Assays were read kinetically using a BioTek Synergy 4 plate reader equipped with Excitation 340/30 nm, Emission1 495/10 nm, Emission2 520/25 nm bandpass filters, a 400 nm dichroic mirror and a Xenon flash lamp set to read Terbium and Fluorescein emission with a 100 μs time delay and 200 μs data collection time. The corrected FRET ratio was calculated as described in the TR-FRET manual (Molecular Devices): ((Em520/Em490)-(P × Em490))/Em490) × 10,000, where P=Em520/Em490 with terbium only, a proportionality factor to correct for terbium donor contribution to acceptor emission. The linear slope of the first 10 min of the reaction was determined as the initial velocity. The resulting velocity versus HsMUB concentration data was fit to a standard one-site binding model and Hill equation in Origin 8 curve-fitting software to obtain nearly identical IC_50_ values, which in this case are equivalent to K_i_ due to conditions of limiting E2 substrate ([S]<<K_m_).

### Lap-bar peptide-inhibition assay

Lap-Bar custom peptide (LeuLysLeuProPheGlyLysThr, Sigma-Aldrich and Peptide 2.0 Inc.), three scrambled peptides (Thr-Leu-Gly-Phe-Lys-Leu-Lys-Pro, Gly-Leu-Thr-Leu-Lys-Pro-Lys-Phe, Thr-Leu-Phe-Lys-Leu-Lys-Gly-Pro; Peptide 2.0 Inc.) and amino acid controls were dissolved in 50 mM Tris-HCl, pH 7.5, 300 mM NaCl and 10% Glycerol. To test inhibition of E2∼Ub formation, 3 μM AtUBC8 was pre-incubated with 5 mM Lap-Bar peptide, control peptides or an equimolar amino acid mix (1 mM Leu, 1 mM Lys and 0.5 mM Pro, Phe, Gly and Thr) and analysed as described above for E2∼Ub-formation assays.

### Data availability

Coordinates and structure factors have been submitted to the Protein Data Bank under accession numbers 4X57 for the AtMUB3:AtUBC8 complex. All additional experimental data are available from the corresponding author on request.

## Additional information

**How to cite this article**: Lu, X. *et al*. A MUB E2 structure reveals E1 selectivity between cognate ubiquitin E2s in eukaryotes. *Nat. Commun.* 7:12580 doi: 10.1038/ncomms12580 (2016).

## Supplementary Material

Supplementary Information Supplementary Figures 1-16 and Supplementary Tables 1-2.

## Figures and Tables

**Figure 1:**
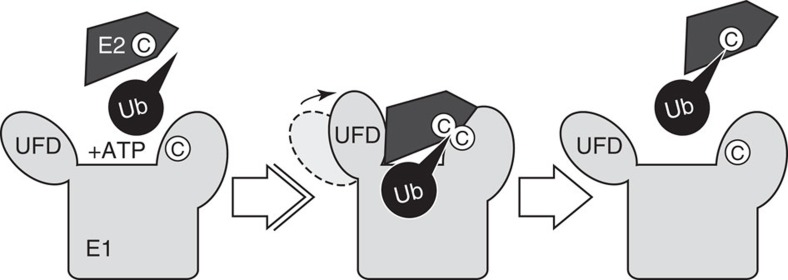
Ubiquitin (Ub) conjugating enzyme (E2) activation by Ub-activating enzyme (E1). Conformational changes are crucial to E1 activity including rotation (closed arrow) of the UFD, which is required to efficiently position E2 for ubiquitin (Ub) thioester (E2∼Ub) formation. Active-site cysteine residues (C, circled) move into proximity for trans-thiolation of Ub from E1 to E2.

**Table 1:** Crystallographic data and refinement statistics.

*Data collection*	
Wavelength (Å)	0.97
Space group	P 63 2 2
Unit cell dimensions (Å)	a=b=135.72c=202.13
Molecules/asymmetric unit	4
Resolution range (Å)	50.00–2.8
Unique observations	27,721
Completeness (%)*	99.8 (100)
R_sym_ (%)	7.2 (7.3)
I/s(I)	11.2 (1.9)
	
*Refinement*	
Resolution (Å)	10–2.8
R_cryst_, R_free_ *	0.22 (0.39), 0.26 (0.36)
Reflections (working/test)	25,543/1,346
Protein atoms	3,720
Sulfate atoms	30
R.m.s.d. bond lengths (Å)	0.01
R.m.s.d. angles (Å^2^)	1.5
<B> protein (Å^2^) :	75
	
*Ramachandran plot*	
Most favoured (%)†	90.7
Allowed (%)	7.8
Generously allowed (%)	1.5

R.m.s.d., root mean-squared deviation.

^*^High-resolution shell for data collection is 2.8–2.85 and for refinement 2.8–2.87.

^†^Calculated by PROCHECK.
