# Supplementary material for: A MUB E2 structure reveals E1 selectivity between cognate ubiquitin E2s in eukaryotes
Source: Nat Commun. 2016 Aug 23;7:12580. doi: 10.1038/ncomms12580 (PMC4996978; doi:10.1038/ncomms12580)
Supplement: Supplementary Information — Supplementary Figures 1-16 and Supplementary Tables 1-2. [file ncomms12580-s1.pdf]

Supplementary Figures

Supplementary Figure 1: Isothermal Titration Calorimetry of AtUBC8 and AtMUB3

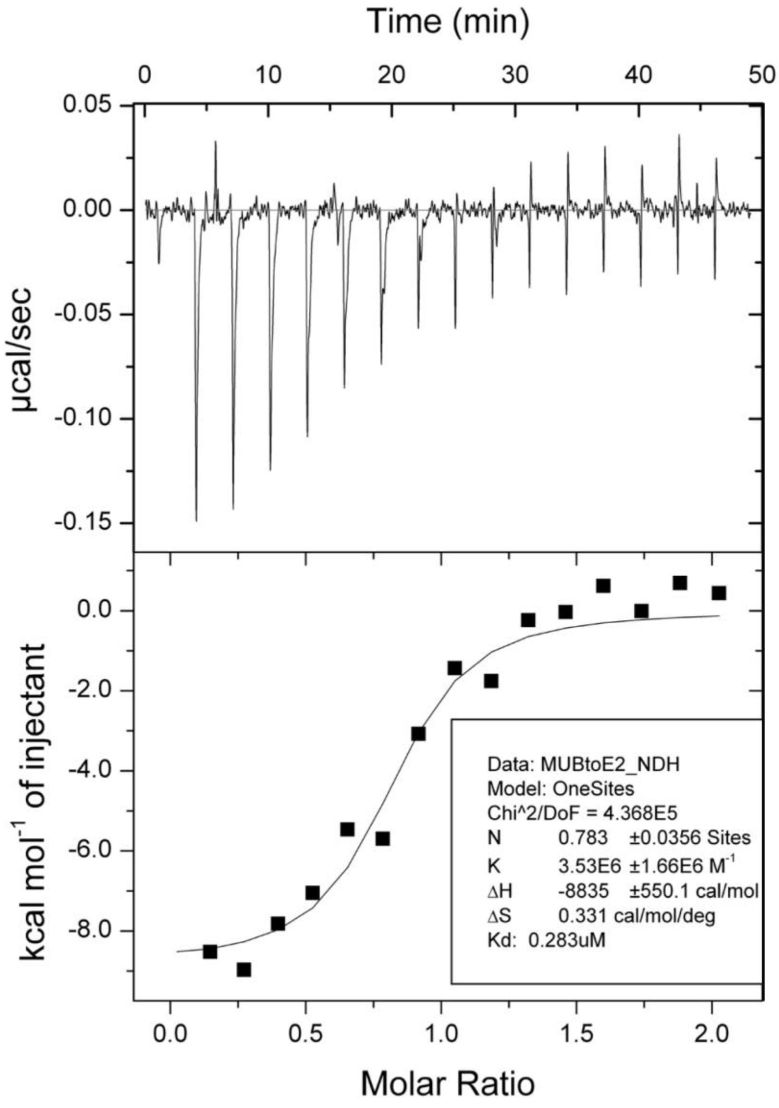

Isothermal titration calorimetry (ITC) was performed on 8 µM AtUBC8 with 110 µM AtMUB3 as the titrant using a MicroCal iTC200 (GE). Proteins were purified as described for crystallization and dialyzed extensively against a buffer containing 25mM HEPES, pH7.5, 200mM NaCl, 1mM TCEP. Data was processed with MicroCal Origin 7 software, and normalized against the dialysis buffer's heat of dilution. This plot is representative of 3 independent experiments, which yielded similar results but were performed at different concentrations of NaCl.

Supplementary Figure 2: Proteins aligned for r.m.s.d determination

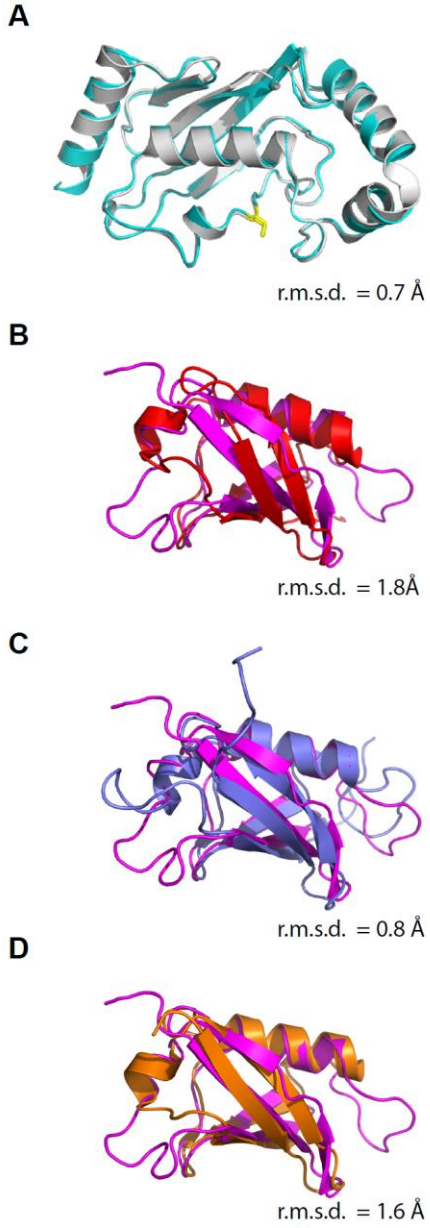

(**a**) AtUBC8 (present study), cyan, is superimposed to Ube2D3 (3UGB), grey.

(**b**) AtMUB3 (present study), magenta, is superimposed to Ub (2FUH), red.

(**c**) AtMUB3 (present study), magenta, is superimposed to AtMUB1 (1SE9), slate.

(**d**) AtMUB3 (present study), magenta, is superimposed to HsSUMO1 (2UYZ), orange.

Supplementary Figure 3: BBS configuration shared by MUB, Ub and SUMO

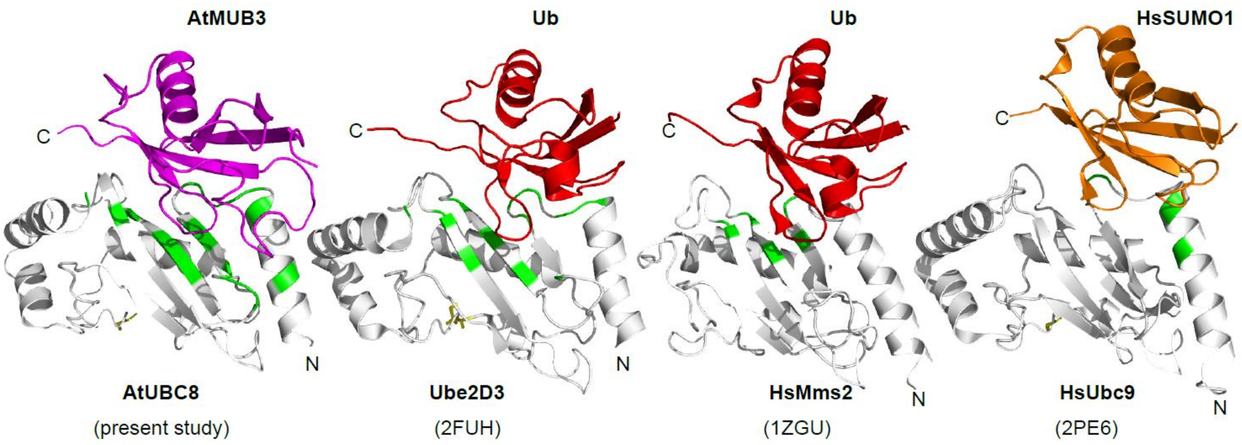

Structures of AtMUB3 (magenta), HsUb (red) and HsSUMO1 (orange) bind at the BBS of respective E2s. All E2s are colored in grey with the binding area colored in green.

Supplementary Figure 4: AtMUB3 interacting vs. non-MUB-interacting Arabidopsis E2s

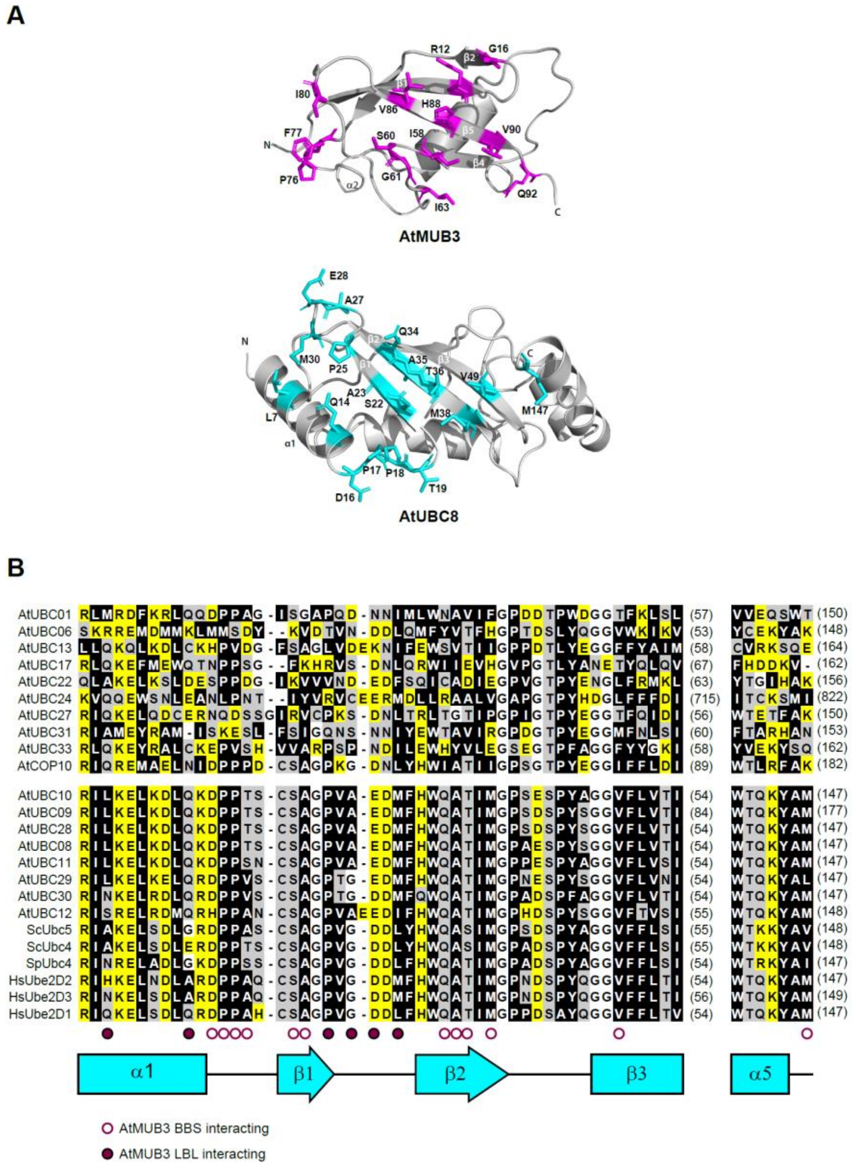

(**a**) The AtMUB3:AtUBC8 complex is presented in open book configuration with interacting residues depicted in stick format. Binding residues are colored magenta in AtMUB3 (Top panel), cyan in AtUBC8 (bottom panel). Secondary structures supporting the interaction residues are indicated.

(**b**) Protein sequence alignment colored as in Fig. 3. Sequences above the space do not interact with MUB by yeast two-hybrid.

Supplementary Figure 5: Stereo view of representative AtMUB3 AtUBC8 electron density

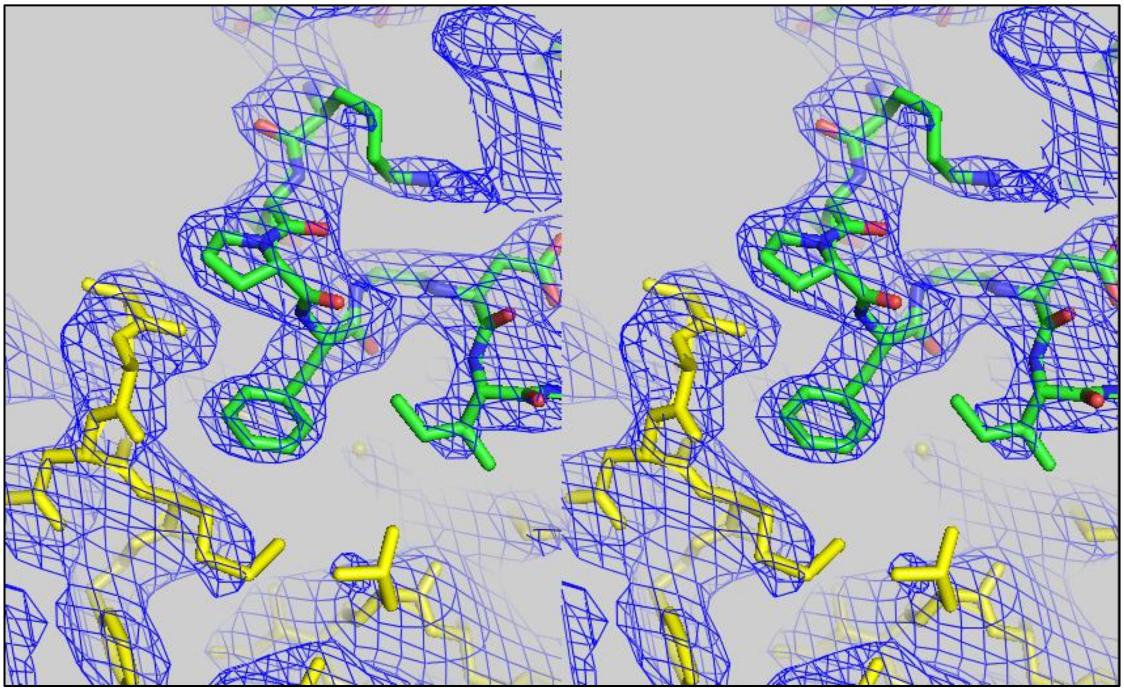

The AtMUB3 LBL (green) with AtUBC8 (yellow) interface represented using a 2Fo-Fc electron density map contoured at 1*sigma* level.

Supplementary Figure 6: MUB LBL construct diagram

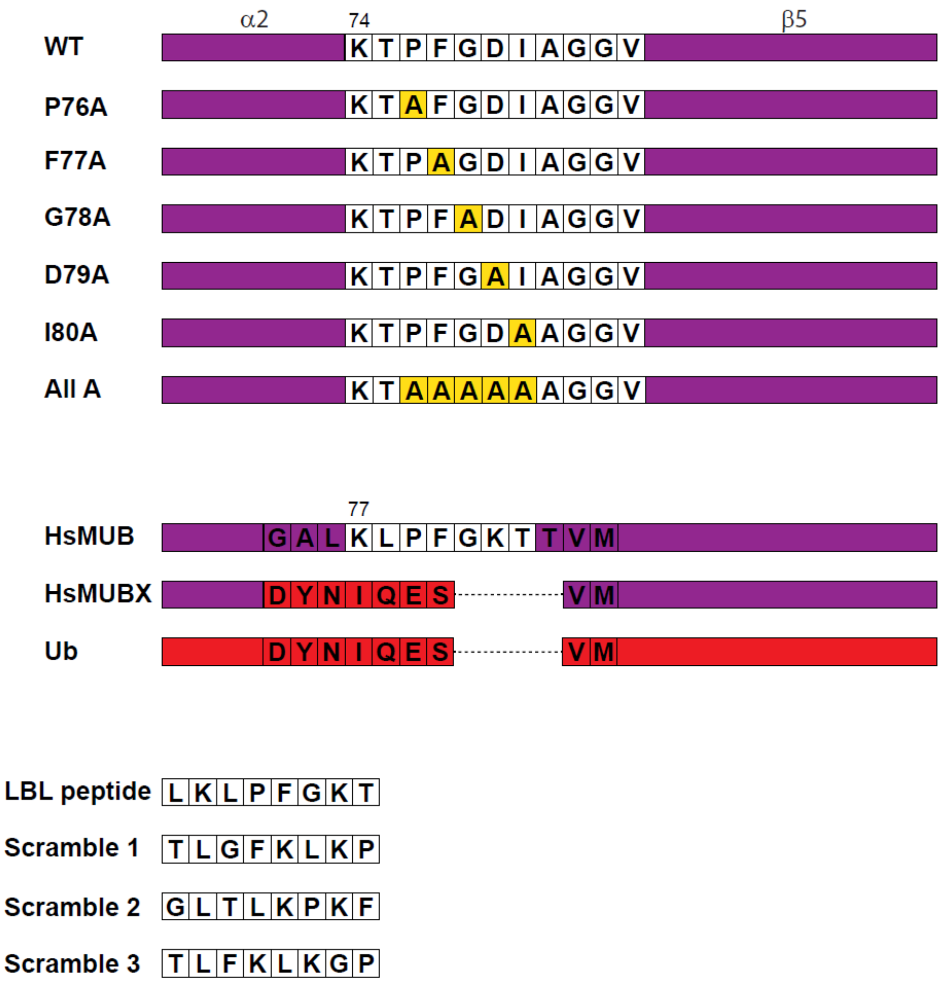

MUB LBL mutants are shown in bar diagrams with LBL detailed and colored in white. Mutated residues are highlighted in yellow. Top panel is AtMUB3 mutants, middle panel is HsMUB mutants with Ub as reference, and bottom panel is LBL and scramble peptides.

Supplementary Figure 7: MUB3 LBL core residues are critical for inhibiting E2~Ub

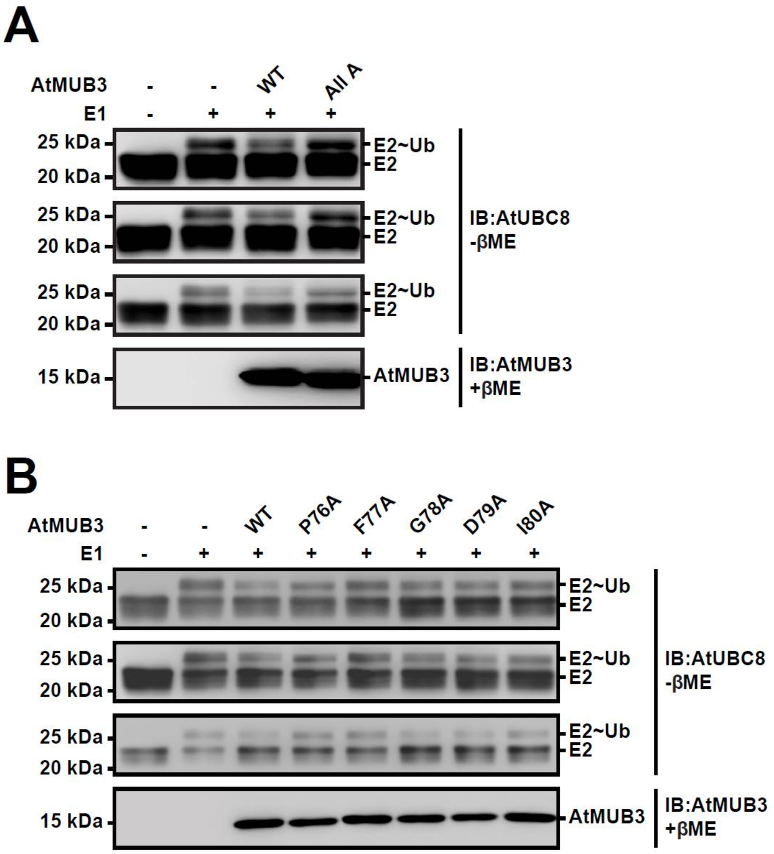

AtUBC8~Ub thioester formation assays exposed to

(**a**) AtMUB3 LBL core quintuple mutant (All A)

(**b**) AtMUB3 LBL core single mutants are shown as immunoblot. Experiments were performed in triplicate prior to quantification of band chemiluminescence.

Supplementary Figure 8: A kinetic model for MUB inhibition of E2 activation

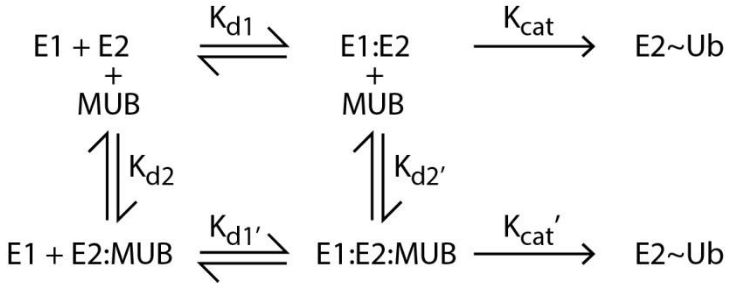

Supplementary Figure 9: Full immunoblots for Figure 2*a*

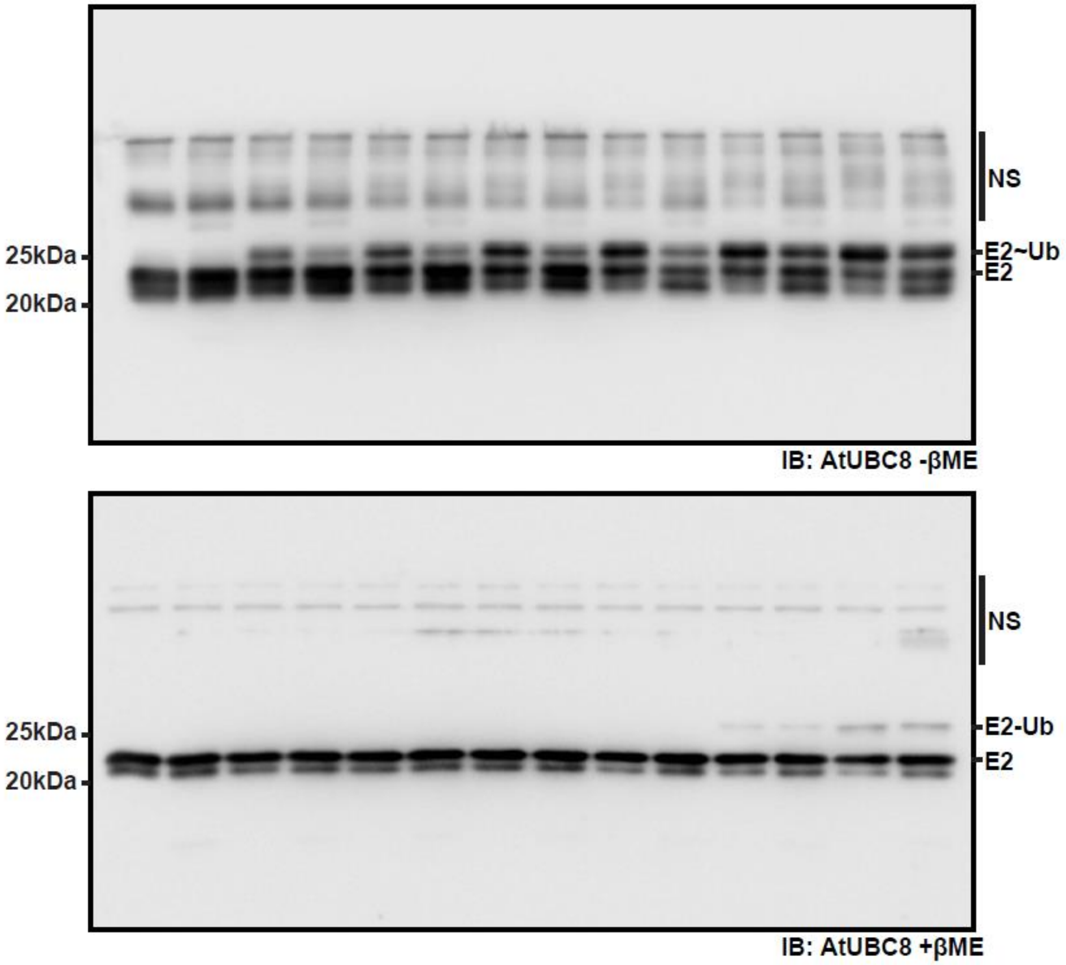

Non-specific bands (NS) correspond to those seen in the leftmost lane, which is minus E1 enzyme.

Supplementary Figure 10: Full immunoblots for Figure 2*b*

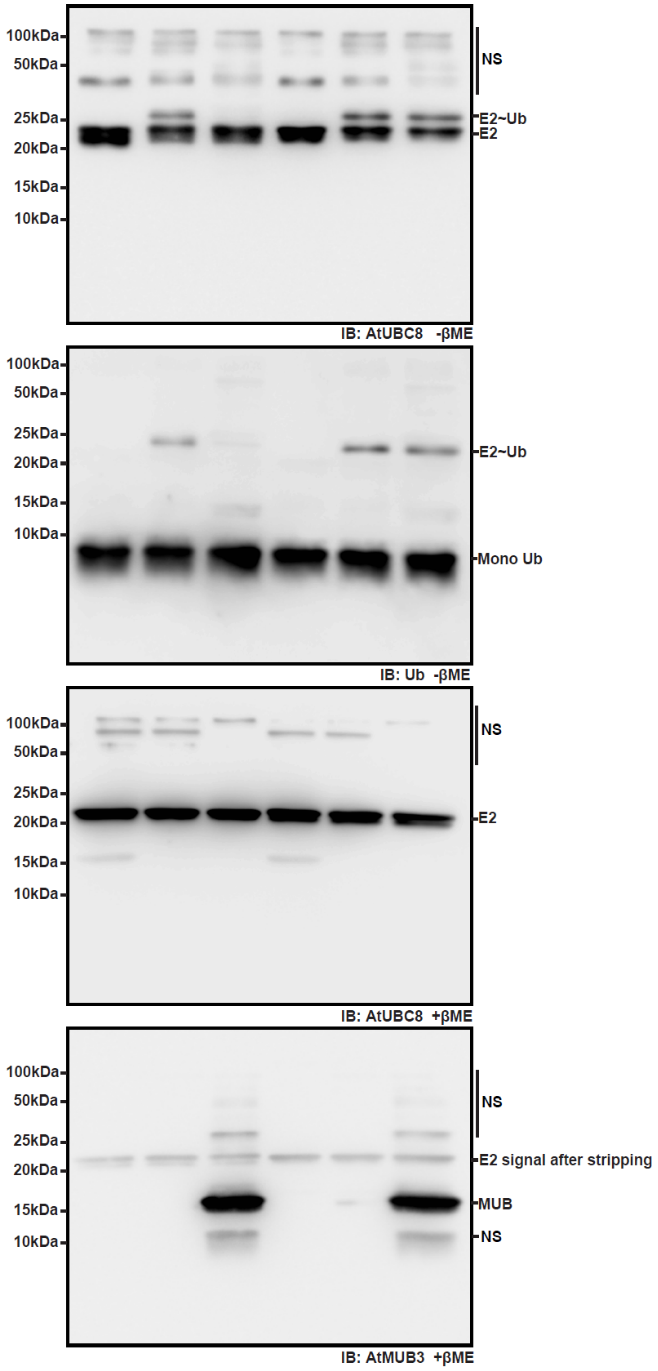

Non-specific bands (NS) correspond to those seen in the leftmost lane, which is minus E1 enzyme, as described.

Supplementary Figure 11: Full immunoblots for Figure 2*c*

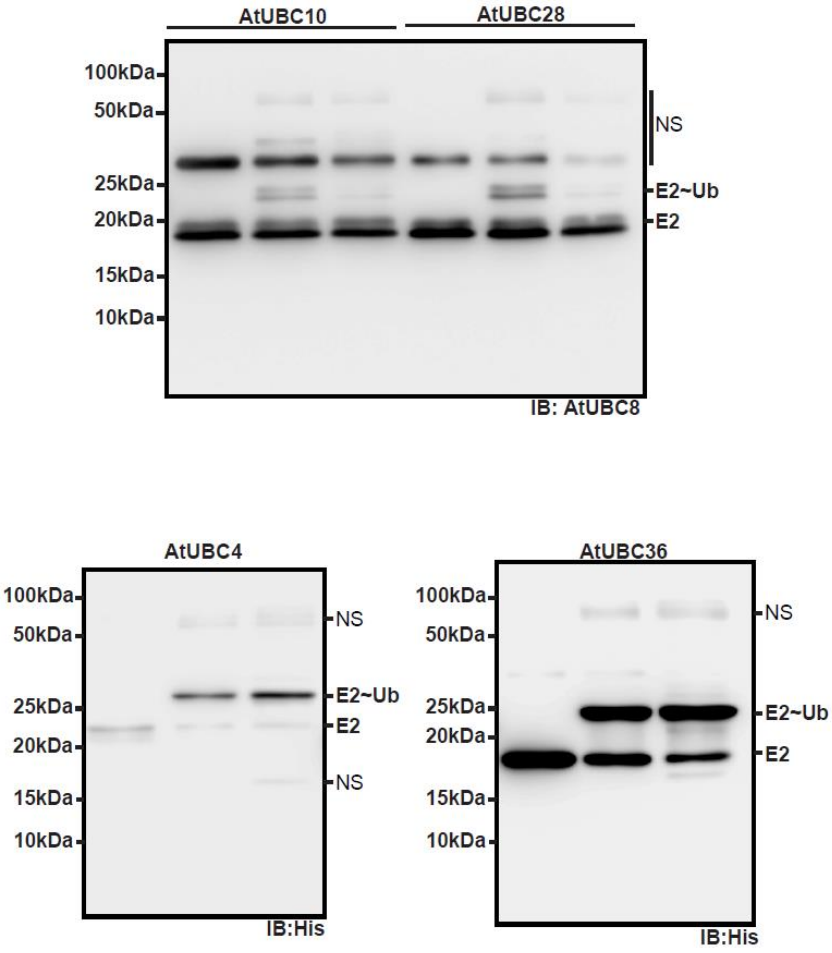

Supplementary Figure 12: Full immunoblots for Figure 3*c*

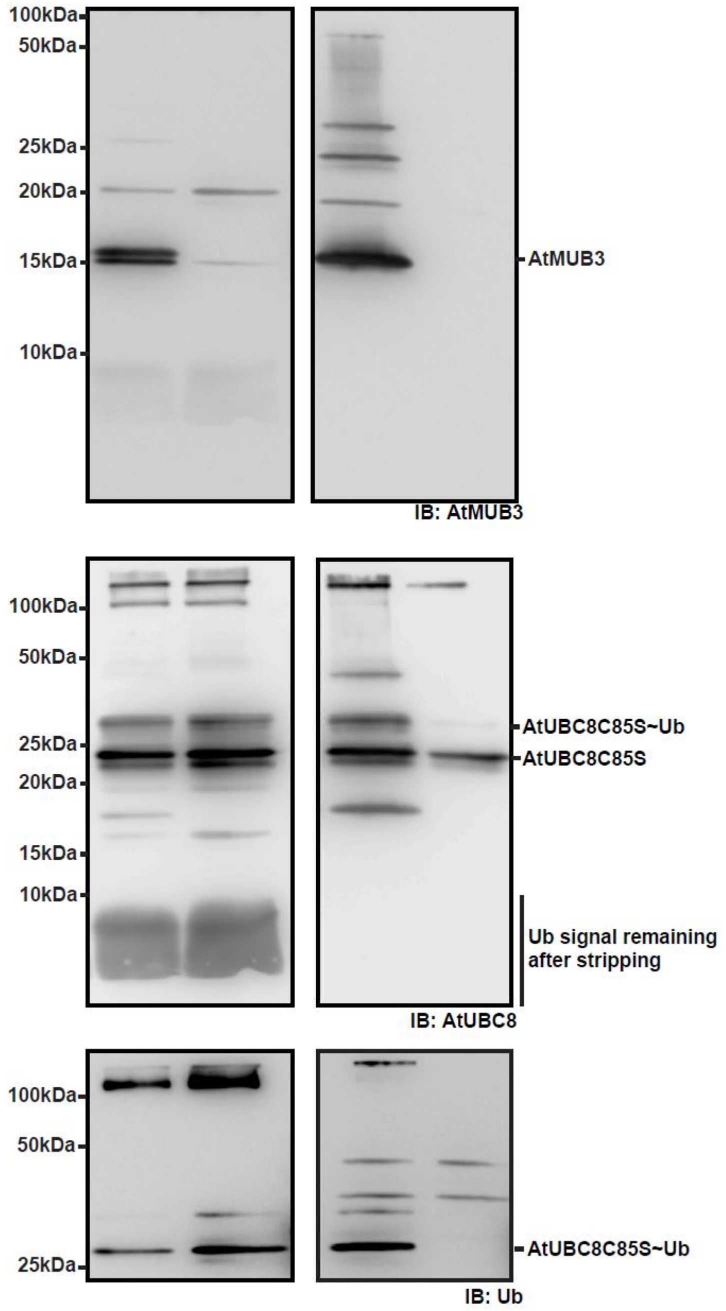

Supplementary Figure 13: Full immunoblots for Figure 3*e*

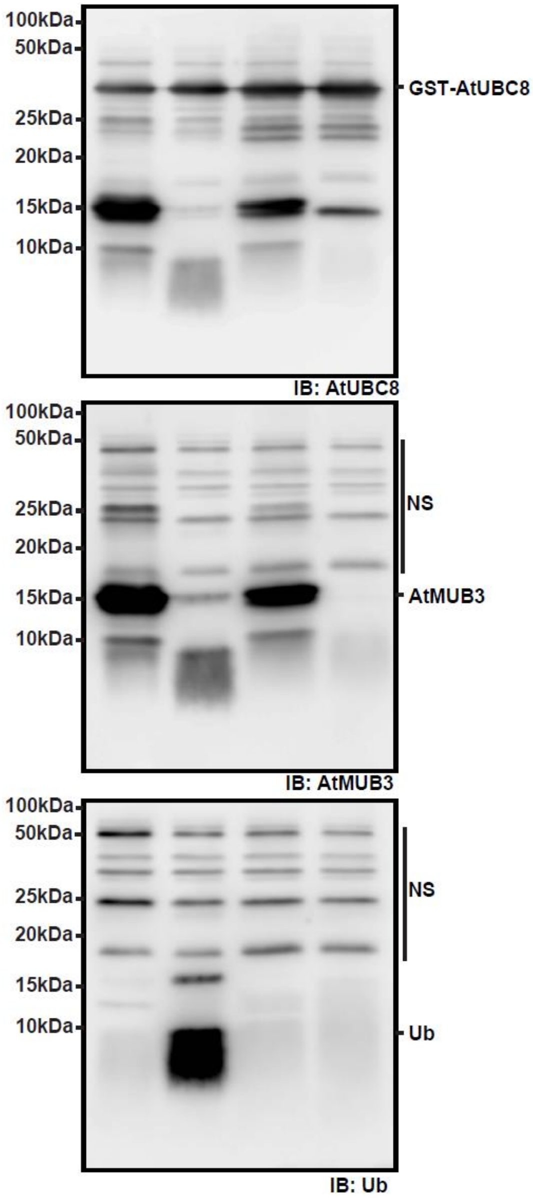

Non-specific (NS) from immunoblot stripping and reprobing.

Supplementary Figure 14: Full immunoblots for Figure 5*a*

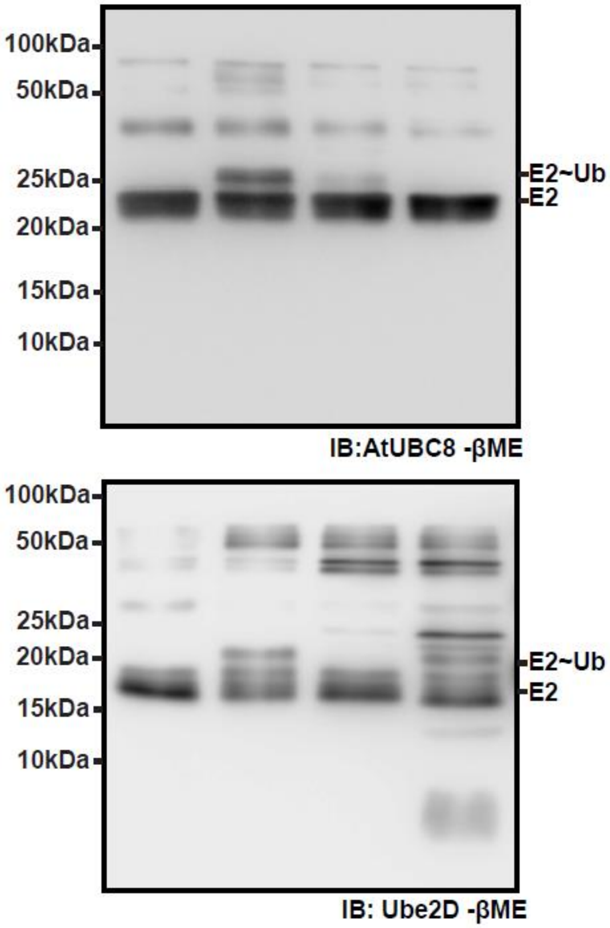

Supplementary Figure 15: Full immunoblots for Figure 5*b*

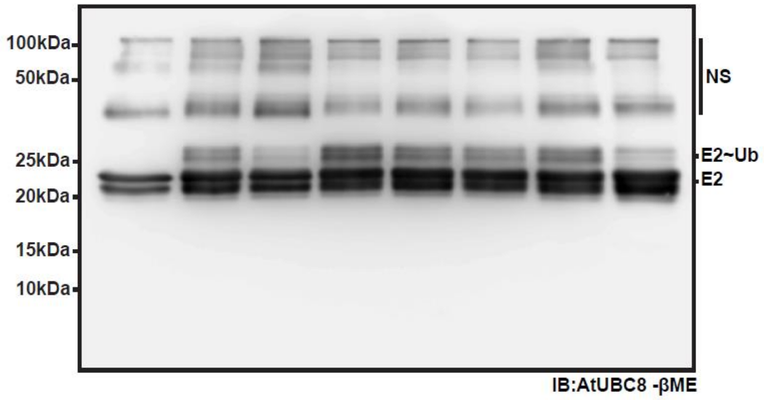

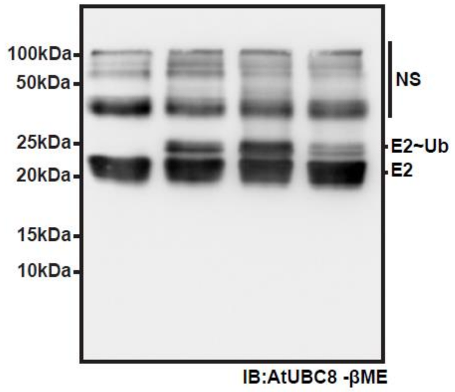

Non-specific bands (NS) correspond to those seen in the leftmost lane, which is minus E1 enzyme.

Supplementary Figure 16: Full immunoblots for Figure 5*d*

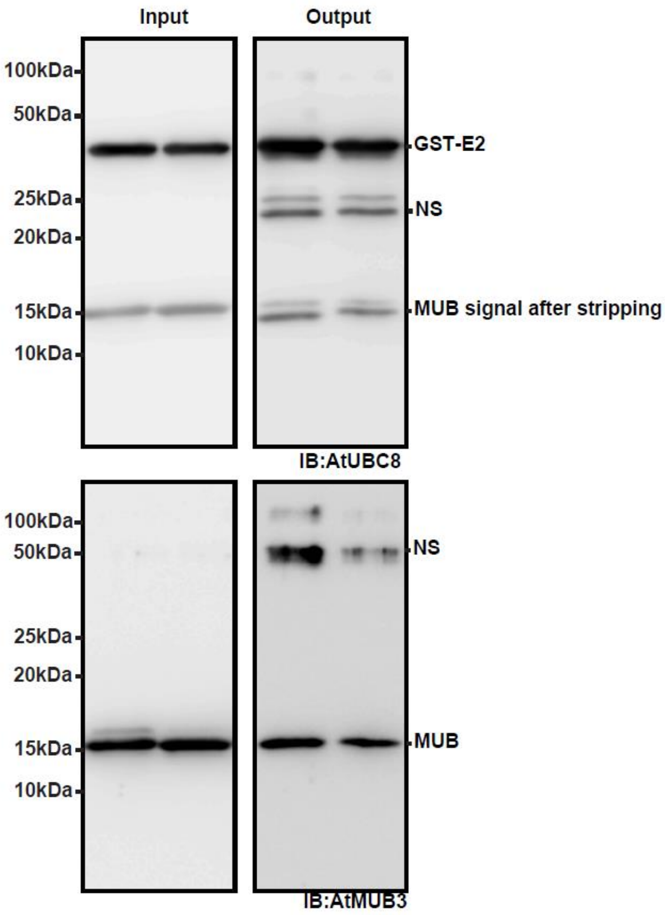

Supplementary Tables

Supplementary Table 1: Surface characteristics of structures shown in Figure S3

	Protein 1	Protein 2	Surface area (Å^2)*	ΔG*
current	AtUBC8	AtMUB3	890.6	-11.7
current	AtUBC8	AtMUB3 (74-82aa)	288.2	-5.0
2FUH	HsUbe2D3	HsUb	547.2	-8.3
1ZGU	HsMMS2	HsUb	624.8	-5.4
2PE6	HsUBC9	HsSUMO1	560.4	-0.2

***** Analysis performed using CCP4 v. 6.5

Supplementary Table 2: Subcloning details and primer sequences

Lab ID	Protein	Plasmid name	entry vector
p72	His-AtMUB3	pET28b+AtMUB3*	
p458	AtMUB3-TEV-HIS	pET28b+AtMUB3TEV	
p469	AtMUB3 P76A-TEV-HIS	pET28b+AtMUB3 P76A	
p460	AtMUB3 F77A-TEV-HIS	pET28b+AtMUB3 F77A	
p470	AtMUB3 G78A-TEV-HIS	pET28b+AtMUB3 G78A	
p471	AtMUB3 D79A-TEV-HIS	pET28b+AtMUB3 D79A	
p472	AtMUB3I80A-TEV-HIS	pET28b+AtMUB3 I80A	
p463	AtMUB3 P76A F77A G78A D79A I80A-TEV-HIS	pET28b+AtMUB3 P76A F77A G78A D79A I80A	
p434	AtMUB3-TEV-His-FLAG	DNA2.0+AtMUB3	
p462	HsMUB-TEV-His	pET28b+HsMUB	
p522	AtUBC8 C85S-TEV-His	p0GWA+AtUBC8 C85S TEV	pentr-D-TOPO+AtUBC8 C85S TEV
p265	His-AtUBC8	pHGWA+AtUBC8*	pentr-D-TOPO+AtUBC8*
p431	AtUBC8-TEV-His	p0GWA+AtUBC8TEV	pentr-D-TOPO+AtUBC8 TEV
p432	AtUBC8 S22R-TEV-His	p0GWA+AtUBC8 S22R TEV	pentr-D-TOPO+AtUBC8 S22R TEV
p269	GST-AtUBC8	pGGWA+AtUBC8*	pentr-D-TOPO+AtUBC8*
p448	His-AtUBC4	pHWGA+AtUBC4*	pDONR201+AtUBC4*
p449	His-AtUBC10	pHWGA+AtUBC10*	pDONR201+AtUBC10*
p453	His-AtUBC28	pHWGA+AtUBC28*	pDONR201+AtUBC28*
p454	His-AtUBC36	pHWGA +AtUBC36*	pDONR201+AtUBC36*
p461	Ube2D3-TEV-His	p0GWA+Ube2D3	pentr-D-TOPO+Ube2D3

**Conventional Cloning**	
**Gateway Cloning**	

Lab ID	5' primer/resriction site	3' primers/resriction site
p72		
p458	CCCTCTAGAAATAATTTTGTTTAACTTTAAGAAGGAGATATACCATGCCGGAGGAAGAATCGAT /XbaI	CTAGCTCGAGTCCCTGAAAATACAGGTTTTCCAAAATGGTGGATGTGCA/XhoI
p469	CCCTCTAGAAATAATTTTGTTTAACTTTAAGAAGGAGATATACCATGCCGGAGGAAGAATCGAT /XbaI	CTAGCTCGAGTCCCTGAAAATACAGGTTTTCCAAAATGGTGGATGTGCA/XhoI
p460	CCCTCTAGAAATAATTTTGTTTAACTTTAAGAAGGAGATATACCATGCCGGAGGAAGAATCGAT /XbaI	CTAGCTCGAGTCCCTGAAAATACAGGTTTTCCAAAATGGTGGATGTGCA/XhoI
p470	CCCTCTAGAAATAATTTTGTTTAACTTTAAGAAGGAGATATACCATGCCGGAGGAAGAATCGAT /XbaI	CTAGCTCGAGTCCCTGAAAATACAGGTTTTCCAAAATGGTGGATGTGCA/XhoI
p471	CCCTCTAGAAATAATTTTGTTTAACTTTAAGAAGGAGATATACCATGCCGGAGGAAGAATCGAT /XbaI	CTAGCTCGAGTCCCTGAAAATACAGGTTTTCCAAAATGGTGGATGTGCA/XhoI
p472	CCCTCTAGAAATAATTTTGTTTAACTTTAAGAAGGAGATATACCATGCCGGAGGAAGAATCGAT /XbaI	CTAGCTCGAGTCCCTGAAAATACAGGTTTTCCAAAATGGTGGATGTGCA/XhoI
p463	CCCTCTAGAAATAATTTTGTTTAACTTTAAGAAGGAGATATACCATGCCGGAGGAAGAATCGAT /XbaI	CTAGCTCGAGTCCCTGAAAATACAGGTTTTCCAAAATGGTGGATGTGCA/XhoI
p434		
p462	ccctctagaaataattttgtttaactttaagaaggagatataccatgtccagtaatgtcccggcg /XbaI	CTAGCTCGAGGTACAGGATTACACAACAATTACTCTCTCCAGTCTTCTC /XhoI
p522	CACCATGGCTTCGAAACGGATCTTG	tccctgaaaatacaggttttcTCCGCCCATGGCATA
p265	CACCATGGCTTCGAAACGGATCTTG	TTAGCCCATGGCATACTTCTGAGTCCA
p431	CACCATGGCTTCGAAACGGATCTTG	tccctgaaaatacaggttttcTCCGCCCATGGCATA
p432	CACCATGGCTTCGAAACGGATCTTG	tccctgaaaatacaggttttcTCCGCCCATGGCATA
p269	CACCATGGCTTCGAAACGGATCTTG	TTAGCCCATGGCATACTTCTGAGTCCA
p448		
p449		
p453		
p454		
p461		

Lab ID	bridging PCR primer forward	bridging PCR primer reverse
p72		
p458		
p469	AAGACAGCATTTGGAGATATTGCTGGTGGAGTCATTGTGATGCATGTTGTTGTACAGCCT	TCCACCAGCAATATCTCCAAATGCTGTCTTACACTGCCCAACAGTCTTGTTGTTCTCCAA
p460	AAGACACCAGCTGGAGATATTGCTGGTGGAGTCATTGTGATGCATGTTGTTGTACAGCCT	TCCACCAGCAATATCTCCAGCTGGTGTCTTACACTGCCCAACAGTCTTGTTGTTCTCCAA
p470	AAGACACCATTTGCAGATATTGCTGGTGGAGTCATTGTGATGCATGTTGTTGTACAGCCT	TCCACCAGCAATATCTGCAAATGGTGTCTTACACTGCCCAACAGTCTTGTTGTTCTCCAA
p471	AAGACACCATTTGGAGCTATTGCTGGTGGAGTCATTGTGATGCATGTTGTTGTACAGCCT	TCCACCAGCAATAGCTCCAAATGGTGTCTTACACTGCCCAACAGTCTTGTTGTTCTCCAA
p472	AAGACACCATTTGGAGATGCTGCTGGTGGAGTCATTGTGATGCATGTTGTTGTACAGCCT	TCCACCAGCAGCATCTCCAAATGGTGTCTTACACTGCCCAACAGTCTTGTTGTTCTCCAA
p463	AAGACAGCAGCTGCAGCTGCTGCTGGTGGAGTCATTGTGATGCATGTTGTTGTACAGCCT	TCCACCAGCAGCAGCTGCAGCTGCTGTCTTACACTGCCCAACAGTCTTGTTGTTCTCCAA
p434		
p462		
p522	TTAGCCTTGACATTTTGAAAGAACAATGGAGCCCTGCCCTCACCATTTCCAAGGTGTTGC	GCAACACCTTGGAAATGGTGAGGGCAGGGCTCCATTGTTCTTTCAAAATGTCAAGGCTAA
p265		
p431		
p432		
p269		
p448		
p449		
p453		
p454		
p461		
